# Hexa­kis­{[1-(dimethyl­amino)­propyl­idene]oxidanium} bis­(dodeca­molybdo­phosphate) *N*,*N*-dimethyl­propionamide penta­solvate

**DOI:** 10.1107/S1600536812036677

**Published:** 2012-08-31

**Authors:** Akbar Raissi Shabari, Mehrdad Pourayoubi, Shadi Derakhshan Rad

**Affiliations:** a Faculty of Chemistry, North Tehran Branch, Islamic Azad University, Tehran, Iran; b Department of Chemistry, Ferdowsi University of Mashhad, Mashhad, Iran

## Abstract

In the asymmetric unit of the title salt, (C_5_H_12_NO)_6_[PMo_12_O_40_]_2_·5C_5_H_11_NO, there are two independent α-Keggin-type [PMo_12_O_40_]^3−^ polyoxidoanions, which show characteristic features with respect to bond lengths and angles. One of the [CH_3_CH_2_C(=OH)N(CH_3_)_2_]^+^ cations is hydrogen bonded to the neighboring polyoxidoanion through a C=O—H⋯O_bridge_ hydrogen bond. The organic mol­ecules and the remaining organic cations form [(C_5_H_11_NO)_2_H]^+^ mol­ecule–cation pairs, two of which lie about inversion centers, through O—H⋯O hydrogen bonds.

## Related literature

For the structure of [CH_3_CH_2_C(=OH)NHCH_3_]_3_[PMo_12_O_40_]·3CH_3_CH_2_C(O)NHCH_3_, and for spectroscopic studies, see: Pourayoubi & Mahjoub (2010 ▶). For an α-Keggin structure, see: Keggin (1933 ▶). For related polyoxidometalate salts containing hydrogen-bonded mol­ecule–cation pairs, see: Pourayoubi & Mahjoub (2010 ▶); Williamson *et al.* (1987 ▶); Hill *et al.* (1988 ▶). For the salt [(QB)_2_H]ClO_4_ (QB: quinoline betaine) containing a mol­ecule–cation pair and for the presence of H atom on an inversion center in the mol­ecule–cation pair, see: Szafran *et al.* (2002 ▶). For very strong hydrogen bonds and this feature as three-centre-four-electron covalent bonds, see: Gilli & Gilli (2000 ▶). For classification of hydrogen bonds and for positive-charge-assisted hydrogen bonds (+CAHB) and for resonance-assisted hydrogen bonds (RAHB), see: Gilli *et al.* (1989 ▶). 
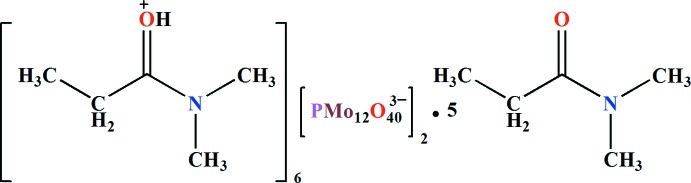


## Experimental

### 

#### Crystal data

(C_5_H_12_NO)_6_[PMo_12_O_40_]_2_·5C_5_H_11_NO
*M* *_r_* = 4763.18
Triclinic, 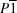

*a* = 11.3691 (5) Å
*b* = 23.7595 (10) Å
*c* = 25.5382 (11) Å
α = 110.322 (1)°
β = 96.172 (1)°
γ = 97.079 (1)°
*V* = 6335.5 (5) Å^3^
*Z* = 2
Mo *K*α radiation
μ = 2.42 mm^−1^
*T* = 100 K
0.14 × 0.13 × 0.11 mm

#### Data collection

Bruker SMART APEXII CCD area-detector diffractometer
Absorption correction: multi-scan (*SADABS*; Bruker, 2008 ▶) *T*_min_ = 0.362, *T*_max_ = 0.433
92777 measured reflections
29585 independent reflections
15934 reflections with *I* > 2σ(*I*)
*R*_int_ = 0.127

#### Refinement

*R*[*F* ^2^ > 2σ(*F* ^2^)] = 0.055
*wR*(*F* ^2^) = 0.091
*S* = 0.94
29585 reflections
1678 parameters
177 restraints
H-atom parameters constrained
Δρ_max_ = 2.17 e Å^−3^
Δρ_min_ = −1.61 e Å^−3^

### 

Data collection: *APEX2* (Bruker, 2009 ▶); cell refinement: *SAINT* (Bruker, 2009 ▶); data reduction: *SAINT*; program(s) used to solve structure: *XS* (Sheldrick, 2008 ▶); program(s) used to refine structure: *XLMP* (Sheldrick, 2008 ▶); molecular graphics: *Mercury* (Macrae *et al.*, 2008 ▶); software used to prepare material for publication: *OLEX2* (Dolomanov *et al.*, 2009 ▶).

## Supplementary Material

Crystal structure: contains datablock(s) I, global. DOI: 10.1107/S1600536812036677/lh5510sup1.cif

Structure factors: contains datablock(s) I. DOI: 10.1107/S1600536812036677/lh5510Isup2.hkl

Additional supplementary materials:  crystallographic information; 3D view; checkCIF report

## Figures and Tables

**Table 1:** Hydrogen-bond geometry (Å, °)

*D*—H⋯*A*	*D*—H	H⋯*A*	*D*⋯*A*	*D*—H⋯*A*
O1*S*—H1*S*⋯O8*S*	1.22	1.22	2.440 (10)	179
O11*S*—H2*S*⋯O2*S*	1.21	1.22	2.426 (11)	179
O3*S*—H3*S*⋯O7*S*	1.21	1.22	2.429 (9)	180
O4*S*—H4*S*⋯O5*S*	1.22	1.23	2.450 (10)	180
O6*S*—H6*S*⋯O8^i^	0.84	1.78	2.606 (8)	170
O9*S*—H9*S*⋯O9*S* ^ii^	1.22	1.22	2.447 (10)	180
O10*S*—H10*S*⋯O10*S* ^iii^	1.22	1.22	2.430 (13)	180

Symmetry codes: (i) 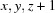; (ii) 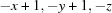; (iii) 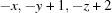.

**Fig. 1.**
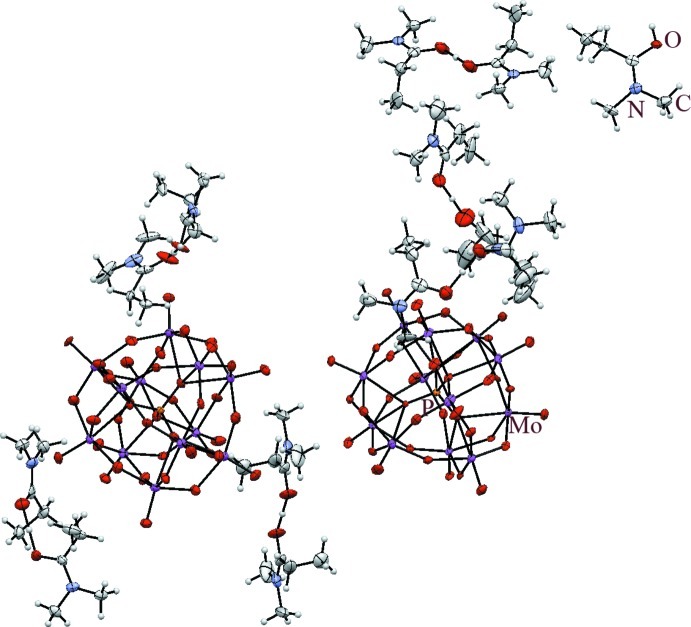
The molecular structure of the title salts showing the organic cations, molecule–cation pairs and inorganic anions. Atoms are represented by 50% probability ellipsoids.

**Fig. 2.**
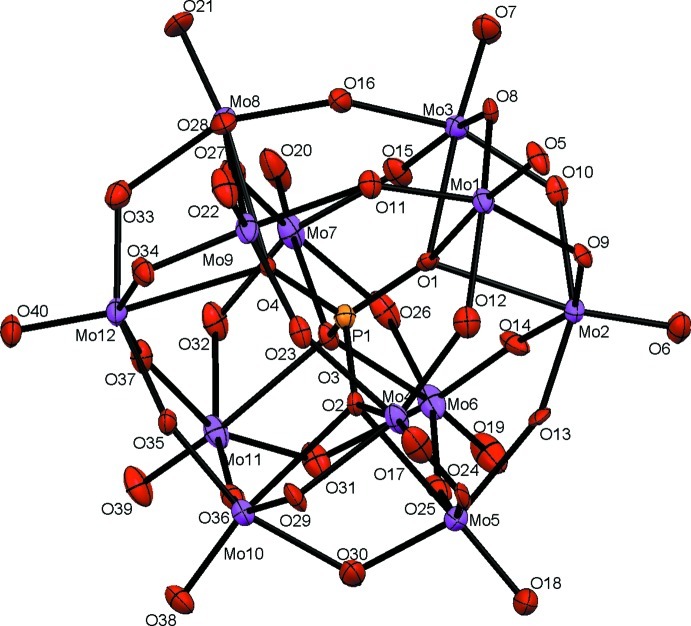
One of the independent Keggin anions of the title salt. Atoms are represented by 50% displacement ellipsoids.

**Fig. 3.**
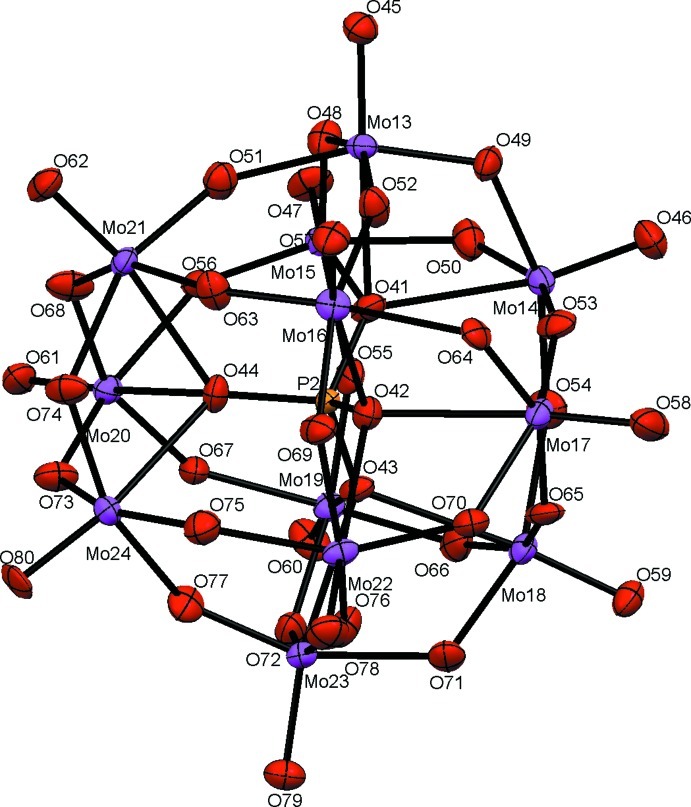
The other independent Keggin anion of the title salt. Atoms are represented by 50% displacement ellipsoids.

**Fig. 4.**
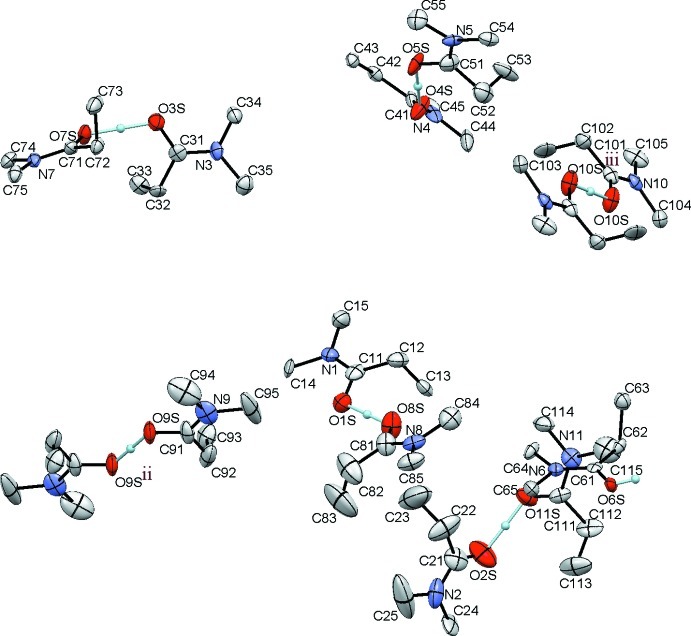
The organic components in the title salt. Atoms are represented by 50% displacement ellipsoids. C—H hydrogen atoms are omitted for clarity. Only symmetry-independent atoms and those linked to them are labeled. Indices ii and iii designate atoms generated by symmetry operations 1 - x, 1 - y, -z and -x, 1 - y, 2 - z, respectively. The hydrogen bonds are listed in Table 1.

**Fig. 5.**
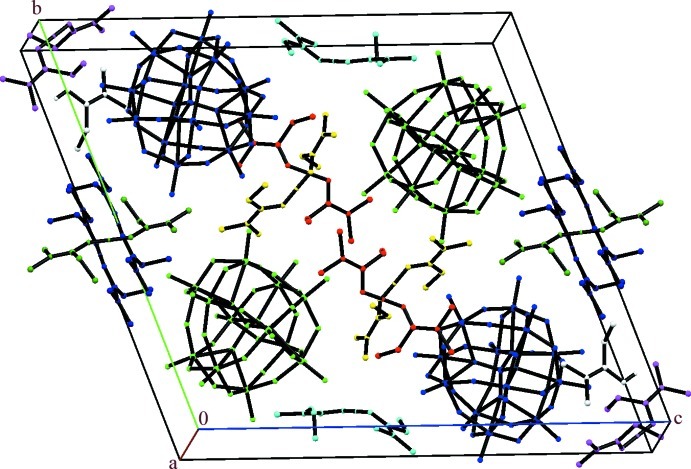
The crystal packing in title salt.

## supplementary crystallographic information

### Comment

In previous work, *N*-methyl-propionamide was used to synthesis a polyoxometalate-based salt, [CH_3_CH_2_C(=OH)NHCH_3_]_3_[PMo_12_O_40_].3CH_3_CH_2_C(O)NHCH_3_, which was studied by X-ray crystallography and spectroscopy (Pourayoubi & Mahjoub, 2010). In the present work, we used *N*,*N*-dimethyl-propionamide to obtain the title salt.

The title salt, [DMPAH]_6_[PMo_12_O_40_]_2_.5DMPA (DMPA = CH_3_CH_2_C(O)N(CH_3_)_2_), (as shown in Fig. 1) contains two symmetrically independent [PMo_12_O_40_]^3-^ polyoxoanions. The anions (polyoxoanion P1 as Fig. 2 and polyoxoanion P2 as Fig. 3) show a classical α-Keggin structure (Keggin, 1933), which consists of a central PO_4_ surrounded by four vertex-sharing Mo_3_O_13_ clusters. Each Mo_3_O_13_ is composed of three MoO_6_ octahedra linked in a triangular arrangement by sharing edges. There are 4 different types of O atoms in each [PMo_12_O_40_]^3-^ anion including 12 terminals, 4 oxygen atoms bonded to P and Mo (O_internal_), 12 MoO_6_ octahedra corner-shared and 12 MoO_6_ octahedra edge-shared oxygen atoms. The P—O bond lengths range from 1.507 (6) to 1.546 (6) Å, and the O—P—O angles are in the range of 108.4 (3) – 110.6 (3)°. The shortest Mo—O bonds are related to the Mo—O_terminal_ bonds with the bond lengths in the range 1.656 (6) [Mo14—O46] to 1.684 (5) Å [Mo2—O6].

One of the [DMPAH]^+^ cation in the crystal (N6/O6S cation) is hydrogen-bonded to the bridging O8 atom of the neighboring P1 polyoxoanion, through a C═O—H···O hydrogen bond (Table 1). All organic molecules in the crystal and the other organic cations form [(DMPA)_2_H]^+^ molecule···cation pair through O—H···O hydrogen bonding. These molecule···cation pairs are similar to those observed for some polyoxometalate salts containing an organic cation and its conjugated base, hydrogen-bonded to each other. Some examples are [NMPAH]_3_[PMo_12_O_40_].3NMPA (NMPA: *N*-methyl-propionamide, Pourayoubi & Mahjoub, 2010), H_3_PMo_12_O_40_.6DMA.CH_3_CN.0.5H_2_O (DMA: *N*,*N*-dimethylacetamide, Williamson *et al.*, 1987) and [(TMU)_2_H]_3_[PW_12_O_40_] (TMU: 1,1,3,3-tetramethylurea, Hill *et al.*, 1988), where [(NMPA)_2_H]^+^, [(DMA)_2_H]^+^ and [(TMU)_2_H]^+^ molecule···cation pairs exist, respectively.

Similar molecule···cation pairs were also observed in the other salts like for example in [(QB)_2_H]ClO_4_ (QB: quinoline betaine, Szafran *et al.*, 2002) where the molecule···cation pair [(QB)_2_H]^+^ exist in the crystal and the H atom is located at the inversion center of molecule···cation pair.

In the title salt, four different molecule···cation pairs (O3S/O7S, O4S/O5S, O1S/O8S, O2S/O11S) and two-half molecule···cation pairs (O9S/O9S^ii^, O10S/O10S^iii^) exist in the asymmetric unit (Fig. 4) (symmetry codes: (ii) –x+1, –y+1, –z; (iii) –x, –y+1, –z+2).

In previously noted reported compounds with molecule···cation pairs, such as in the title compound, very strong homoconjugated O—H···O hydrogen bonds exist (homoconjugated: the same H-donor and H-acceptor atoms). In the O—H···O hydrogen bonds of the title salt, the O···O distances are 2.440 (10), 2.426 (11), 2.429 (9), 2.450 (10), 2.447 (10) and 2.430 (13) Å. The short O···O distances in these hydrogen bonds as well as the symmetry of the molecule···cation pairs O9S/O9S^ii^ and O10S/O10S^iii^ in the title salt support the idea that the very strong hydrogen bonds involves essentially a feature of three-centre-four-electron covalent bonds (Gilli & Gilli, 2000).

The presence of a positive-charge centered on the atoms involving in hydrogen bonding in each of the molecule···cation pairs, [C_5_H_11_NOHONH_11_C_5_]^+^, leads to the observed very strong hydrogen bond. Considering the classification proposed by Gilli and co-workers (1989), the hydrogen bond in this example is of the type positive-charge-assisted hydrogen bond (+CAHB) and also of the type resonance assisted hydrogen bond (RAHB). This means that two mechanisms help to strength of hydrogen bond in the structure of title salt and related structures. The overview of crystal packing in the title salt is given in Fig. 5.

### Experimental

Synthesis of the title salt: A solution of *N*,*N*-dimethyl-propionamide (4.94 mmol) in H_2_O (10 ml)/HCl (5 ml, 1 *M*) was added to a solution of H_3_PMo_12_O_40_ (0.82 mmol) in H_2_O (10 ml) and stirred. The solid formed was solved by adding CH_3_CN (10 ml) and stirred (3 h). After filtering, the solution was hold in a beaker at room temperature. It takes few days to obtain the yellow crystals from this solution.

IR (cm^-1^): 756, 868, 952, 1059, 1179, 1232, 1261, 1381, 1408, 1443, 1578, 1677, 1929, 2945, 3475. ^31^P-NMR ((D_6_)DMSO): -3.56. ^1^H-NMR ((D_6_)DMSO): 0.95 (*t*), 2.26 (*q*), 2.79 (*s*), 2.94 (*s*). ^13^C-NMR ((D_6_)DMSO): 10.13 (*s*), 26.43 (*s*), 35.67 (*s*), 37.43 (*s*), 173.55 (*s*). UV-Vis (H_2_O/CH_3_CN, λ_max_): 222 nm, 256 nm.

### Refinement

The H-atom positions were calculated, and the H atoms were refined in riding model, except for central H atoms in (C_5_H_11_NO)_2_H cations. Those were found in difference Fourier synthesis but then placed in the center of the O···O line and not refined. SIMU instructions were applied to organic components 2, 11, 8 and 6 to stabilize the refinement.

### Crystal data

**Table:**

(C_5_H_12_NO)_6_[PMo_12_O_40_]_2_·5C_5_H_11_NO	*Z* = 2
*M**_r_* = 4763.18	*F*(000) = 4600
Triclinic, *P*1	*D*_x_ = 2.497 Mg m^−^^3^
*a* = 11.3691 (5) Å	Mo *K*α radiation, λ = 0.71073 Å
*b* = 23.7595 (10) Å	Cell parameters from 5907 reflections
*c* = 25.5382 (11) Å	θ = 2.2–25.4°
α = 110.322 (1)°	µ = 2.42 mm^−^^1^
β = 96.172 (1)°	*T* = 100 K
γ = 97.079 (1)°	Block, colourless
*V* = 6335.5 (5) Å^3^	0.14 × 0.13 × 0.11 mm

### Data collection

**Table:**

Bruker SMART APEXII CCD area-detector diffractometer	29585 independent reflections
Radiation source: sealed tube	15934 reflections with *I* > 2σ(*I*)
Graphite monochromator	*R*_int_ = 0.127
Detector resolution: 8 pixels mm^-1^	θ_max_ = 27.7°, θ_min_ = 1.0°
φ and ω scans	*h* = −14→14
Absorption correction: multi-scan (*SADABS*; Bruker, 2008)	*k* = −31→31
*T*_min_ = 0.362, *T*_max_ = 0.433	*l* = −33→33
92777 measured reflections	

### Refinement

**Table:**

Refinement on *F*^2^	Primary atom site location: structure-invariant direct methods
Least-squares matrix: full	Secondary atom site location: difference Fourier map
*R*[*F*^2^ > 2σ(*F*^2^)] = 0.055	Hydrogen site location: inferred from neighbouring sites
*wR*(*F*^2^) = 0.091	H-atom parameters constrained
*S* = 0.94	*w* = 1/[σ^2^(*F*_o_^2^) + (0.0179*P*)^2^] where *P* = (*F*_o_^2^ + 2*F*_c_^2^)/3
29585 reflections	(Δ/σ)_max_ = 0.001
1678 parameters	Δρ_max_ = 2.17 e Å^−^^3^
177 restraints	Δρ_min_ = −1.61 e Å^−^^3^

### Special details

**Table:**

Experimental. Absorption correction: SADABS-2008/1 (Bruker,2008) was used for absorption correction. R(int) was 0.0734 before and 0.0592 after correction. The Ratio of minimum to maximum transmission is 0.8358. The λ/2 correction factor is 0.0015.
Geometry. All e.s.d.'s (except the e.s.d. in the dihedral angle between two l.s. planes) are estimated using the full covariance matrix. The cell e.s.d.'s are taken into account individually in the estimation of e.s.d.'s in distances, angles and torsion angles; correlations between e.s.d.'s in cell parameters are only used when they are defined by crystal symmetry. An approximate (isotropic) treatment of cell e.s.d.'s is used for estimating e.s.d.'s involving l.s. planes.
Refinement. Refinement of *F*^2^ against ALL reflections. The weighted *R*-factor *wR* and goodness of fit *S* are based on *F*^2^, conventional *R*-factors *R* are based on *F*, with *F* set to zero for negative *F*^2^. The threshold expression of *F*^2^ > σ(*F*^2^) is used only for calculating *R*-factors(gt) *etc*. and is not relevant to the choice of reflections for refinement. *R*-factors based on *F*^2^ are statistically about twice as large as those based on *F*, and *R*- factors based on ALL data will be even larger.

### Fractional atomic coordinates and isotropic or equivalent isotropic displacement parameters (Å^2^)

**Table:**

	*x*	*y*	*z*	*U*_iso_*/*U*_eq_	
Mo1	0.72133 (7)	0.23008 (3)	0.09526 (3)	0.01466 (17)	
Mo2	0.69051 (7)	0.14511 (3)	0.17754 (3)	0.01517 (17)	
Mo3	0.44040 (7)	0.17853 (3)	0.11661 (3)	0.01620 (17)	
Mo4	0.94075 (7)	0.33452 (3)	0.21935 (3)	0.01814 (18)	
Mo5	0.87016 (7)	0.26272 (3)	0.30924 (3)	0.02027 (19)	
Mo6	0.55090 (8)	0.20280 (4)	0.30706 (3)	0.0227 (2)	
Mo7	0.33363 (7)	0.26137 (4)	0.24982 (4)	0.0227 (2)	
Mo8	0.40785 (7)	0.34093 (4)	0.15184 (3)	0.02085 (19)	
Mo9	0.70113 (7)	0.39526 (3)	0.15455 (3)	0.01827 (18)	
Mo10	0.84075 (7)	0.41122 (3)	0.34161 (3)	0.01637 (17)	
Mo11	0.53720 (7)	0.35451 (4)	0.36374 (3)	0.02035 (19)	
Mo12	0.57153 (7)	0.44749 (3)	0.27315 (3)	0.01681 (18)	
P1	0.6333 (2)	0.29689 (10)	0.22934 (10)	0.0127 (5)	
O1	0.6240 (4)	0.2384 (2)	0.1769 (2)	0.0107 (12)	
O2	0.7627 (5)	0.3170 (2)	0.2615 (2)	0.0127 (13)	
O3	0.5500 (5)	0.2841 (2)	0.2694 (2)	0.0142 (13)	
O4	0.5949 (5)	0.3474 (2)	0.2105 (2)	0.0116 (12)	
O5	0.7850 (5)	0.2114 (2)	0.0369 (2)	0.0185 (14)	
O6	0.7196 (5)	0.0749 (2)	0.1688 (2)	0.0204 (14)	
O7	0.3278 (5)	0.1343 (3)	0.0653 (2)	0.0229 (15)	
O8	0.5619 (5)	0.1888 (2)	0.0663 (2)	0.0149 (13)	
O9	0.7473 (5)	0.1595 (2)	0.1177 (2)	0.0127 (13)	
O10	0.5268 (5)	0.1233 (2)	0.1299 (2)	0.0151 (13)	
O11	0.6914 (5)	0.3064 (2)	0.1057 (2)	0.0148 (13)	
O12	0.8630 (5)	0.2704 (2)	0.1548 (2)	0.0178 (14)	
O13	0.8112 (5)	0.1943 (2)	0.2334 (2)	0.0167 (14)	
O14	0.5887 (5)	0.1577 (2)	0.2391 (2)	0.0198 (14)	
O15	0.3692 (5)	0.1983 (2)	0.1793 (2)	0.0194 (14)	
O16	0.4197 (5)	0.2606 (2)	0.1168 (2)	0.0175 (14)	
O17	1.0723 (5)	0.3537 (3)	0.2017 (2)	0.0239 (15)	
O18	0.9649 (6)	0.2305 (3)	0.3400 (2)	0.0288 (16)	
O19	0.5444 (6)	0.1545 (3)	0.3414 (3)	0.0360 (18)	
O20	0.1867 (5)	0.2377 (3)	0.2435 (3)	0.0348 (18)	
O21	0.2821 (5)	0.3478 (3)	0.1160 (3)	0.0318 (17)	
O22	0.7521 (6)	0.4294 (3)	0.1123 (2)	0.0244 (15)	
O23	0.8386 (5)	0.3849 (2)	0.1920 (2)	0.0150 (13)	
O24	0.9773 (5)	0.2893 (2)	0.2617 (2)	0.0185 (14)	
O25	0.7262 (5)	0.2385 (2)	0.3268 (2)	0.0215 (15)	
O26	0.3944 (5)	0.2024 (3)	0.2834 (2)	0.0245 (16)	
O27	0.3424 (5)	0.3117 (3)	0.2103 (2)	0.0208 (14)	
O28	0.5270 (5)	0.3738 (3)	0.1220 (2)	0.0208 (14)	
O29	0.9499 (5)	0.4045 (2)	0.2928 (2)	0.0190 (14)	
O30	0.9006 (5)	0.3422 (2)	0.3585 (2)	0.0210 (14)	
O31	0.5458 (5)	0.2810 (3)	0.3705 (2)	0.0233 (15)	
O32	0.3709 (5)	0.3192 (3)	0.3214 (2)	0.0234 (15)	
O33	0.4387 (5)	0.4201 (3)	0.2167 (2)	0.0209 (14)	
O34	0.6651 (5)	0.4577 (2)	0.2128 (2)	0.0180 (14)	
O35	0.7374 (5)	0.4461 (2)	0.3085 (2)	0.0144 (13)	
O36	0.6975 (5)	0.3805 (2)	0.3697 (2)	0.0176 (14)	
O37	0.5186 (5)	0.4106 (2)	0.3194 (2)	0.0182 (14)	
O38	0.9102 (5)	0.4691 (2)	0.4003 (2)	0.0219 (15)	
O39	0.5041 (6)	0.3975 (3)	0.4262 (2)	0.0277 (16)	
O40	0.5618 (5)	0.5206 (2)	0.3055 (2)	0.0213 (15)	
Mo13	−0.01411 (7)	0.75602 (4)	0.12341 (3)	0.01869 (18)	
Mo14	0.05746 (8)	0.64854 (4)	0.17670 (3)	0.0227 (2)	
Mo15	−0.14922 (7)	0.73780 (4)	0.23164 (3)	0.01855 (18)	
Mo16	0.29055 (7)	0.84651 (4)	0.15632 (3)	0.01929 (18)	
Mo17	0.36894 (7)	0.72440 (4)	0.18694 (3)	0.01857 (18)	
Mo18	0.27086 (7)	0.69722 (4)	0.31071 (3)	0.02190 (19)	
Mo19	0.03895 (7)	0.76343 (4)	0.36725 (3)	0.01807 (18)	
Mo20	−0.04571 (7)	0.89458 (4)	0.33453 (3)	0.01835 (18)	
Mo21	0.04318 (7)	0.91848 (3)	0.21820 (3)	0.01747 (18)	
Mo22	0.46619 (7)	0.87020 (4)	0.28276 (3)	0.01958 (19)	
Mo23	0.32199 (7)	0.84765 (4)	0.39867 (3)	0.02066 (19)	
Mo24	0.24239 (7)	0.96452 (3)	0.34206 (3)	0.01913 (18)	
P2	0.1580 (2)	0.80597 (10)	0.26081 (10)	0.0140 (5)	
O41	0.0557 (5)	0.7583 (3)	0.2179 (2)	0.0223 (15)	
O42	0.2696 (5)	0.8098 (3)	0.2344 (2)	0.0219 (15)	
O43	0.1847 (5)	0.7870 (3)	0.3120 (2)	0.0195 (14)	
O44	0.1160 (5)	0.8681 (3)	0.2805 (2)	0.0205 (14)	
O45	−0.0865 (5)	0.7467 (3)	0.0596 (2)	0.0251 (15)	
O46	0.0393 (6)	0.5746 (3)	0.1394 (3)	0.0308 (17)	
O47	−0.2914 (5)	0.7105 (3)	0.2309 (3)	0.0289 (16)	
O48	−0.1549 (5)	0.7425 (3)	0.1607 (2)	0.0236 (15)	
O49	0.0069 (5)	0.6782 (3)	0.1144 (2)	0.0238 (15)	
O50	−0.0915 (6)	0.6577 (3)	0.1972 (3)	0.0290 (16)	
O51	−0.0251 (5)	0.8432 (3)	0.1678 (3)	0.0258 (15)	
O52	0.1400 (5)	0.7859 (3)	0.1229 (3)	0.0260 (16)	
O53	0.2176 (5)	0.6785 (3)	0.1628 (2)	0.0256 (16)	
O54	0.1377 (6)	0.6561 (3)	0.2451 (3)	0.0292 (16)	
O55	−0.0695 (5)	0.7306 (2)	0.3021 (2)	0.0223 (15)	
O56	−0.1352 (5)	0.8184 (3)	0.2729 (2)	0.0238 (15)	
O57	0.3255 (5)	0.8628 (3)	0.1004 (2)	0.0255 (15)	
O58	0.4570 (6)	0.6757 (3)	0.1572 (3)	0.0350 (18)	
O59	0.3182 (5)	0.6374 (3)	0.3202 (2)	0.0245 (15)	
O60	−0.0419 (6)	0.7513 (3)	0.4149 (2)	0.0285 (16)	
O61	−0.1554 (5)	0.9230 (3)	0.3674 (2)	0.0267 (16)	
O62	−0.0031 (5)	0.9639 (3)	0.1861 (3)	0.0254 (15)	
O63	0.1939 (5)	0.8994 (3)	0.1867 (3)	0.0249 (15)	
O64	0.3578 (5)	0.7692 (3)	0.1413 (2)	0.0212 (15)	
O65	0.3576 (5)	0.7097 (3)	0.2596 (2)	0.0270 (16)	
O66	0.1294 (5)	0.7022 (3)	0.3534 (3)	0.0279 (16)	
O67	−0.0022 (5)	0.8435 (3)	0.3692 (2)	0.0218 (15)	
O68	−0.0595 (5)	0.9238 (3)	0.2697 (2)	0.0277 (16)	
O69	0.4257 (5)	0.8837 (3)	0.2093 (2)	0.0244 (15)	
O70	0.4914 (5)	0.7943 (3)	0.2390 (2)	0.0233 (15)	
O71	0.3536 (5)	0.7615 (3)	0.3734 (2)	0.0237 (15)	
O72	0.1830 (5)	0.8186 (3)	0.4200 (3)	0.0260 (16)	
O73	0.0822 (5)	0.9567 (3)	0.3676 (2)	0.0270 (16)	
O74	0.1639 (5)	0.9791 (3)	0.2828 (2)	0.0262 (16)	
O75	0.3669 (5)	0.9354 (3)	0.3088 (2)	0.0280 (16)	
O76	0.4309 (5)	0.8521 (3)	0.3443 (3)	0.0274 (16)	
O77	0.2737 (5)	0.9144 (3)	0.3900 (2)	0.0265 (16)	
O78	0.6041 (5)	0.9106 (3)	0.3034 (3)	0.0303 (16)	
O79	0.4197 (5)	0.8763 (3)	0.4590 (2)	0.0232 (15)	
O80	0.3013 (5)	1.0344 (2)	0.3872 (2)	0.0255 (16)	
O1S	0.2901 (6)	0.3481 (3)	0.4770 (3)	0.0419 (19)	
H1S	0.3240	0.3322	0.5158	0.080*	
O8S	0.3560 (7)	0.3154 (3)	0.5544 (3)	0.055 (2)	
N1	0.2340 (7)	0.4258 (4)	0.4589 (4)	0.037 (2)	
N8	0.3482 (7)	0.2594 (4)	0.6071 (4)	0.038 (2)	
C11	0.2805 (9)	0.4042 (6)	0.4955 (5)	0.039 (3)	
C12	0.3294 (10)	0.4441 (5)	0.5549 (4)	0.045 (3)	
H12A	0.3064	0.4230	0.5806	0.054*	
H12B	0.2946	0.4820	0.5647	0.054*	
C13	0.4663 (9)	0.4602 (4)	0.5633 (4)	0.035 (3)	
H13A	0.4889	0.4836	0.5399	0.053*	
H13B	0.5005	0.4227	0.5521	0.053*	
H13C	0.4971	0.4846	0.6032	0.053*	
C14	0.1940 (9)	0.3893 (5)	0.3993 (4)	0.046 (3)	
H14A	0.2471	0.4029	0.3767	0.068*	
H14B	0.1117	0.3941	0.3882	0.068*	
H14C	0.1962	0.3464	0.3930	0.068*	
C15	0.2204 (10)	0.4908 (5)	0.4740 (4)	0.055 (4)	
H15A	0.1488	0.4976	0.4919	0.083*	
H15B	0.2121	0.5006	0.4397	0.083*	
H15C	0.2915	0.5170	0.5004	0.083*	
C81	0.3378 (10)	0.2637 (5)	0.5574 (5)	0.044 (3)	
C82	0.3108 (12)	0.2080 (6)	0.5055 (6)	0.089 (5)	
H82A	0.2308	0.2070	0.4851	0.107*	
H82B	0.3047	0.1722	0.5171	0.107*	
C83	0.3983 (15)	0.2008 (7)	0.4647 (6)	0.131 (7)	
H83A	0.4801	0.2089	0.4853	0.197*	
H83B	0.3910	0.2298	0.4454	0.197*	
H83C	0.3809	0.1592	0.4366	0.197*	
C84	0.3755 (10)	0.3143 (5)	0.6583 (5)	0.054 (3)	
H84A	0.3005	0.3256	0.6710	0.080*	
H84B	0.4195	0.3476	0.6501	0.080*	
H84C	0.4249	0.3064	0.6880	0.080*	
C85	0.3248 (10)	0.2015 (5)	0.6167 (5)	0.054 (3)	
H85A	0.3849	0.1765	0.6020	0.081*	
H85B	0.2445	0.1795	0.5971	0.081*	
H85C	0.3295	0.2100	0.6574	0.081*	
O2S	0.9384 (9)	0.3753 (4)	0.5486 (4)	0.078 (3)	
H2S	0.9168	0.4136	0.5908	0.080*	
O11S	0.8933 (7)	0.4517 (3)	0.6321 (3)	0.045 (2)	
N2	0.8958 (10)	0.2814 (5)	0.4872 (4)	0.065 (3)	
N11	0.8570 (8)	0.4922 (4)	0.7196 (4)	0.038 (2)	
C115	0.8773 (12)	0.5060 (6)	0.7788 (5)	0.075 (4)	
H11A	0.8470	0.4699	0.7868	0.113*	
H11B	0.8353	0.5393	0.7975	0.113*	
H11C	0.9636	0.5184	0.7932	0.113*	
C21	0.8649 (11)	0.3274 (7)	0.5264 (6)	0.056 (3)	
C22	0.7446 (10)	0.3217 (7)	0.5404 (6)	0.079 (4)	
H22A	0.7505	0.3451	0.5814	0.095*	
H22B	0.7176	0.2783	0.5344	0.095*	
C23	0.6500 (12)	0.3412 (7)	0.5103 (7)	0.127 (7)	
H23A	0.6728	0.3845	0.5169	0.190*	
H23B	0.5744	0.3347	0.5242	0.190*	
H23C	0.6397	0.3174	0.4697	0.190*	
C24	1.0256 (10)	0.2917 (6)	0.4748 (4)	0.068 (4)	
H24A	1.0320	0.3204	0.4551	0.102*	
H24B	1.0449	0.2528	0.4510	0.102*	
H24C	1.0820	0.3083	0.5105	0.102*	
C25	0.8014 (17)	0.2265 (6)	0.4619 (6)	0.139 (8)	
H25A	0.7797	0.2117	0.4915	0.208*	
H25B	0.8316	0.1947	0.4332	0.208*	
H25C	0.7304	0.2368	0.4443	0.208*	
C111	0.9274 (9)	0.4614 (5)	0.6828 (5)	0.033 (2)	
C112	1.0393 (9)	0.4482 (5)	0.7081 (5)	0.047 (3)	
H11D	1.0886	0.4869	0.7348	0.056*	
H11E	1.0183	0.4223	0.7300	0.056*	
C113	1.1105 (10)	0.4176 (5)	0.6660 (6)	0.075 (4)	
H11F	1.1899	0.4173	0.6850	0.112*	
H11G	1.1196	0.4392	0.6399	0.112*	
H11H	1.0697	0.3756	0.6448	0.112*	
C114	0.7478 (9)	0.5091 (5)	0.6974 (5)	0.046 (3)	
H11I	0.6795	0.4947	0.7125	0.069*	
H11J	0.7329	0.4903	0.6560	0.069*	
H11K	0.7582	0.5535	0.7089	0.069*	
O3S	0.1649 (6)	1.0010 (3)	0.0704 (3)	0.0377 (18)	
H3S	0.1105	0.9680	0.0243	0.080*	
O7S	0.0554 (6)	0.9348 (3)	−0.0220 (3)	0.0326 (17)	
N3	0.3262 (7)	1.0145 (3)	0.1342 (3)	0.029 (2)	
N7	−0.0353 (7)	0.8406 (4)	−0.0761 (3)	0.0260 (19)	
C31	0.2717 (9)	0.9925 (4)	0.0810 (4)	0.028 (2)	
C32	0.3302 (9)	0.9564 (4)	0.0322 (4)	0.036 (3)	
H32A	0.2811	0.9154	0.0126	0.043*	
H32B	0.4107	0.9514	0.0471	0.043*	
C33	0.3423 (9)	0.9883 (4)	−0.0098 (4)	0.038 (3)	
H33A	0.3658	0.9608	−0.0441	0.057*	
H33B	0.4037	1.0251	0.0072	0.057*	
H33C	0.2652	0.9996	−0.0196	0.057*	
C34	0.2673 (9)	1.0462 (4)	0.1799 (4)	0.034 (3)	
H34A	0.3018	1.0896	0.1948	0.051*	
H34B	0.2785	1.0297	0.2100	0.051*	
H34C	0.1813	1.0408	0.1661	0.051*	
C35	0.4488 (9)	1.0068 (5)	0.1525 (4)	0.041 (3)	
H35A	0.4449	0.9705	0.1625	0.061*	
H35B	0.4875	1.0427	0.1855	0.061*	
H35C	0.4955	1.0022	0.1216	0.061*	
C71	0.0111 (9)	0.8812 (4)	−0.0267 (4)	0.027 (2)	
C72	0.0047 (8)	0.8669 (5)	0.0264 (4)	0.031 (2)	
H72A	0.0772	0.8893	0.0547	0.037*	
H72B	0.0030	0.8228	0.0173	0.037*	
C73	−0.1067 (9)	0.8850 (5)	0.0511 (4)	0.047 (3)	
H73A	−0.1780	0.8659	0.0220	0.070*	
H73B	−0.1004	0.9294	0.0644	0.070*	
H73C	−0.1137	0.8715	0.0830	0.070*	
C74	−0.0320 (8)	0.8563 (4)	−0.1264 (4)	0.030 (2)	
H74A	0.0211	0.8955	−0.1165	0.046*	
H74B	−0.1132	0.8593	−0.1413	0.046*	
H74C	−0.0018	0.8246	−0.1553	0.046*	
C75	−0.0922 (8)	0.7797 (4)	−0.0864 (4)	0.029 (2)	
H75A	−0.0309	0.7542	−0.0862	0.044*	
H75B	−0.1443	0.7633	−0.1234	0.044*	
H75C	−0.1405	0.7800	−0.0568	0.044*	
O4S	0.1803 (6)	0.9051 (4)	0.5716 (3)	0.055 (2)	
H4S	0.0937	0.8960	0.5939	0.080*	
O5S	0.0065 (6)	0.8873 (4)	0.6162 (3)	0.043 (2)	
N4	0.3051 (8)	0.9698 (4)	0.5510 (4)	0.042 (2)	
N5	−0.0413 (7)	0.8891 (4)	0.6984 (3)	0.0291 (19)	
C41	0.1967 (9)	0.9403 (5)	0.5457 (4)	0.034 (3)	
C42	0.0950 (8)	0.9475 (4)	0.5063 (4)	0.025 (2)	
H42A	0.0860	0.9909	0.5192	0.031*	
H42B	0.1163	0.9360	0.4678	0.031*	
C43	−0.0218 (8)	0.9100 (4)	0.5034 (4)	0.026 (2)	
H43A	−0.0535	0.9276	0.5387	0.039*	
H43B	−0.0103	0.8683	0.4982	0.039*	
H43C	−0.0788	0.9095	0.4715	0.039*	
C44	0.4054 (9)	0.9608 (6)	0.5864 (4)	0.059 (4)	
H44A	0.3959	0.9179	0.5824	0.089*	
H44B	0.4067	0.9862	0.6260	0.089*	
H44C	0.4810	0.9722	0.5745	0.089*	
C45	0.3334 (9)	1.0136 (5)	0.5239 (5)	0.069 (5)	
H45A	0.2631	1.0324	0.5190	0.104*	
H45B	0.3555	0.9926	0.4869	0.104*	
H45C	0.4007	1.0452	0.5477	0.104*	
C51	0.0382 (10)	0.8948 (5)	0.6683 (5)	0.040 (2)	
C52	0.1759 (10)	0.9037 (5)	0.6905 (5)	0.051 (3)	
H52A	0.1893	0.8879	0.7214	0.061*	
H52B	0.2182	0.8821	0.6596	0.061*	
C53	0.2180 (12)	0.9684 (5)	0.7109 (4)	0.056 (4)	
H53A	0.1811	0.9886	0.7439	0.084*	
H53B	0.1962	0.9839	0.6810	0.084*	
H53C	0.3056	0.9766	0.7218	0.084*	
C54	−0.0139 (9)	0.8924 (4)	0.7572 (4)	0.034 (3)	
H54A	0.0315	0.8603	0.7584	0.051*	
H54B	−0.0890	0.8869	0.7719	0.051*	
H54C	0.0340	0.9323	0.7806	0.051*	
C55	−0.1661 (9)	0.8691 (5)	0.6717 (4)	0.042 (3)	
H55A	−0.1840	0.8245	0.6561	0.064*	
H55B	−0.1807	0.8847	0.6412	0.064*	
H55C	−0.2180	0.8846	0.6998	0.064*	
O6S	0.4522 (6)	0.1312 (3)	0.9627 (2)	0.0248 (15)	
H6S	0.4845	0.1535	0.9958	0.037*	
N6	0.3678 (7)	0.1372 (4)	0.8833 (3)	0.0280 (19)	
C61	0.4317 (8)	0.1653 (4)	0.9331 (4)	0.023 (2)	
C62	0.4769 (8)	0.2310 (4)	0.9568 (4)	0.024 (2)	
H62A	0.5007	0.2445	0.9263	0.029*	
H62B	0.5493	0.2395	0.9854	0.029*	
C63	0.3825 (9)	0.2677 (4)	0.9844 (4)	0.037 (3)	
H63A	0.3485	0.2498	1.0099	0.055*	
H63B	0.3184	0.2665	0.9549	0.055*	
H63C	0.4207	0.3101	1.0059	0.055*	
C64	0.3332 (8)	0.1684 (5)	0.8437 (4)	0.031 (3)	
H64A	0.3858	0.2080	0.8553	0.046*	
H64B	0.2496	0.1744	0.8448	0.046*	
H64C	0.3415	0.1432	0.8052	0.046*	
C65	0.3190 (9)	0.0721 (4)	0.8614 (4)	0.041 (3)	
H65A	0.3380	0.0552	0.8907	0.061*	
H65B	0.3546	0.0513	0.8282	0.061*	
H65C	0.2316	0.0659	0.8507	0.061*	
O9S	0.4656 (6)	0.5029 (3)	0.0445 (3)	0.048 (2)	
H9S	0.5000	0.5000	0.0000	0.080*	
N9	0.4940 (10)	0.5380 (4)	0.1375 (4)	0.051 (3)	
C91	0.5348 (11)	0.5310 (5)	0.0906 (5)	0.041 (3)	
C92	0.6632 (10)	0.5556 (5)	0.0893 (5)	0.057 (4)	
H92A	0.7116	0.5653	0.1269	0.069*	
H92B	0.6962	0.5235	0.0614	0.069*	
C93	0.6760 (10)	0.6105 (5)	0.0746 (5)	0.052 (3)	
H93A	0.6325	0.6007	0.0364	0.079*	
H93B	0.7612	0.6249	0.0759	0.079*	
H93C	0.6429	0.6424	0.1018	0.079*	
C94	0.3699 (13)	0.5122 (6)	0.1356 (6)	0.085 (5)	
H94A	0.3266	0.5451	0.1540	0.128*	
H94B	0.3681	0.4836	0.1555	0.128*	
H94C	0.3316	0.4908	0.0962	0.128*	
C95	0.5637 (15)	0.5731 (5)	0.1947 (5)	0.100 (6)	
H95A	0.6064	0.5461	0.2082	0.151*	
H95B	0.5089	0.5902	0.2208	0.151*	
H95C	0.6218	0.6062	0.1929	0.151*	
O10S	0.0936 (6)	0.5344 (3)	1.0069 (3)	0.044 (2)	
H10S	0.0000	0.5000	1.0000	0.080*	
N10	0.1840 (8)	0.6249 (4)	1.0125 (3)	0.029 (2)	
C101	0.0843 (9)	0.5848 (5)	1.0014 (4)	0.030 (2)	
C102	−0.0320 (9)	0.5967 (5)	0.9819 (4)	0.037 (3)	
H10A	−0.0359	0.6403	1.0012	0.045*	
H10B	−0.0963	0.5724	0.9922	0.045*	
C103	−0.0528 (11)	0.5812 (5)	0.9195 (5)	0.064 (4)	
H10C	−0.0446	0.5386	0.9003	0.096*	
H10D	0.0064	0.6078	0.9096	0.096*	
H10E	−0.1338	0.5869	0.9075	0.096*	
C104	0.3008 (9)	0.6084 (5)	1.0276 (4)	0.038 (3)	
H10F	0.2881	0.5760	1.0429	0.057*	
H10G	0.3532	0.6443	1.0560	0.057*	
H10H	0.3387	0.5941	0.9937	0.057*	
C105	0.1869 (10)	0.6819 (5)	1.0047 (4)	0.048 (3)	
H10I	0.2218	0.7153	1.0404	0.071*	
H10J	0.1050	0.6866	0.9927	0.071*	
H10K	0.2360	0.6828	0.9756	0.071*	

### Atomic displacement parameters (Å^2^)

**Table:**

	*U*^11^	*U*^22^	*U*^33^	*U*^12^	*U*^13^	*U*^23^
Mo1	0.0181 (4)	0.0128 (4)	0.0127 (4)	0.0040 (3)	0.0041 (3)	0.0034 (3)
Mo2	0.0197 (4)	0.0106 (4)	0.0148 (4)	0.0045 (3)	0.0016 (3)	0.0039 (3)
Mo3	0.0141 (4)	0.0145 (4)	0.0173 (4)	0.0026 (3)	0.0009 (3)	0.0029 (3)
Mo4	0.0144 (4)	0.0152 (4)	0.0203 (5)	0.0018 (3)	0.0052 (4)	0.0005 (4)
Mo5	0.0265 (5)	0.0160 (4)	0.0166 (4)	0.0085 (4)	−0.0024 (4)	0.0039 (4)
Mo6	0.0373 (5)	0.0151 (4)	0.0176 (5)	0.0020 (4)	0.0103 (4)	0.0075 (4)
Mo7	0.0169 (4)	0.0204 (4)	0.0262 (5)	0.0012 (4)	0.0110 (4)	0.0013 (4)
Mo8	0.0211 (5)	0.0181 (4)	0.0214 (5)	0.0095 (4)	−0.0011 (4)	0.0041 (4)
Mo9	0.0291 (5)	0.0134 (4)	0.0154 (4)	0.0065 (4)	0.0074 (4)	0.0071 (3)
Mo10	0.0186 (4)	0.0129 (4)	0.0151 (4)	0.0033 (3)	0.0018 (3)	0.0020 (3)
Mo11	0.0274 (5)	0.0166 (4)	0.0164 (5)	0.0031 (4)	0.0108 (4)	0.0033 (4)
Mo12	0.0203 (4)	0.0131 (4)	0.0176 (4)	0.0069 (3)	0.0051 (4)	0.0042 (3)
P1	0.0143 (11)	0.0110 (11)	0.0137 (12)	0.0026 (9)	0.0032 (9)	0.0051 (9)
O1	0.008 (3)	0.012 (3)	0.012 (3)	0.001 (2)	−0.001 (2)	0.005 (3)
O2	0.015 (3)	0.010 (3)	0.012 (3)	0.006 (2)	0.003 (3)	0.001 (3)
O3	0.013 (3)	0.014 (3)	0.014 (3)	0.004 (3)	0.007 (3)	0.001 (3)
O4	0.018 (3)	0.009 (3)	0.008 (3)	0.007 (2)	0.001 (2)	0.002 (2)
O5	0.030 (4)	0.013 (3)	0.012 (3)	0.003 (3)	0.012 (3)	0.002 (3)
O6	0.018 (3)	0.013 (3)	0.029 (4)	0.003 (3)	0.002 (3)	0.007 (3)
O7	0.016 (3)	0.020 (3)	0.026 (4)	0.002 (3)	0.000 (3)	0.001 (3)
O8	0.021 (3)	0.008 (3)	0.014 (3)	0.007 (3)	0.002 (3)	0.000 (3)
O9	0.016 (3)	0.012 (3)	0.008 (3)	0.004 (3)	0.005 (2)	0.000 (2)
O10	0.016 (3)	0.015 (3)	0.016 (3)	0.004 (3)	0.011 (3)	0.004 (3)
O11	0.018 (3)	0.014 (3)	0.015 (3)	0.004 (3)	0.007 (3)	0.006 (3)
O12	0.019 (3)	0.017 (3)	0.020 (4)	0.003 (3)	0.008 (3)	0.008 (3)
O13	0.023 (4)	0.019 (3)	0.011 (3)	0.013 (3)	−0.002 (3)	0.007 (3)
O14	0.030 (4)	0.016 (3)	0.009 (3)	−0.002 (3)	−0.001 (3)	0.003 (3)
O15	0.014 (3)	0.018 (3)	0.020 (4)	−0.002 (3)	0.004 (3)	0.001 (3)
O16	0.019 (3)	0.019 (3)	0.013 (3)	0.007 (3)	0.001 (3)	0.002 (3)
O17	0.012 (3)	0.025 (4)	0.028 (4)	0.000 (3)	0.011 (3)	0.000 (3)
O18	0.041 (4)	0.019 (4)	0.021 (4)	0.014 (3)	−0.007 (3)	0.002 (3)
O19	0.070 (5)	0.019 (4)	0.020 (4)	−0.003 (4)	0.010 (4)	0.010 (3)
O20	0.024 (4)	0.025 (4)	0.039 (5)	0.001 (3)	0.012 (3)	−0.009 (3)
O21	0.026 (4)	0.031 (4)	0.031 (4)	0.017 (3)	−0.011 (3)	0.003 (3)
O22	0.041 (4)	0.017 (3)	0.019 (4)	0.006 (3)	0.018 (3)	0.006 (3)
O23	0.019 (3)	0.010 (3)	0.016 (3)	0.001 (3)	0.011 (3)	0.004 (3)
O24	0.017 (3)	0.013 (3)	0.017 (3)	0.009 (3)	0.001 (3)	−0.006 (3)
O25	0.040 (4)	0.014 (3)	0.009 (3)	0.004 (3)	0.001 (3)	0.003 (3)
O26	0.026 (4)	0.021 (3)	0.021 (4)	−0.003 (3)	0.016 (3)	0.000 (3)
O27	0.016 (3)	0.019 (3)	0.024 (4)	0.003 (3)	0.004 (3)	0.003 (3)
O28	0.028 (4)	0.019 (3)	0.015 (3)	0.009 (3)	−0.003 (3)	0.006 (3)
O29	0.022 (3)	0.013 (3)	0.015 (3)	0.002 (3)	0.004 (3)	−0.005 (3)
O30	0.018 (3)	0.017 (3)	0.023 (4)	0.004 (3)	−0.001 (3)	0.002 (3)
O31	0.036 (4)	0.016 (3)	0.019 (4)	0.001 (3)	0.014 (3)	0.007 (3)
O32	0.023 (4)	0.021 (3)	0.024 (4)	0.005 (3)	0.016 (3)	0.002 (3)
O33	0.024 (4)	0.025 (4)	0.018 (4)	0.013 (3)	0.007 (3)	0.009 (3)
O34	0.028 (4)	0.010 (3)	0.016 (3)	0.003 (3)	0.004 (3)	0.005 (3)
O35	0.015 (3)	0.011 (3)	0.016 (3)	0.006 (3)	0.004 (3)	0.002 (3)
O36	0.026 (4)	0.014 (3)	0.011 (3)	0.002 (3)	0.002 (3)	0.003 (3)
O37	0.019 (3)	0.014 (3)	0.023 (4)	0.005 (3)	0.010 (3)	0.005 (3)
O38	0.029 (4)	0.016 (3)	0.015 (3)	−0.001 (3)	0.002 (3)	0.000 (3)
O39	0.036 (4)	0.023 (4)	0.021 (4)	0.005 (3)	0.018 (3)	0.001 (3)
O40	0.028 (4)	0.013 (3)	0.023 (4)	0.009 (3)	0.007 (3)	0.006 (3)
Mo13	0.0172 (4)	0.0218 (4)	0.0145 (4)	0.0039 (4)	−0.0013 (3)	0.0044 (4)
Mo14	0.0344 (5)	0.0132 (4)	0.0175 (5)	0.0034 (4)	−0.0022 (4)	0.0041 (4)
Mo15	0.0150 (4)	0.0188 (4)	0.0210 (5)	0.0001 (3)	0.0025 (4)	0.0073 (4)
Mo16	0.0171 (4)	0.0222 (4)	0.0198 (5)	0.0010 (4)	0.0055 (4)	0.0092 (4)
Mo17	0.0197 (4)	0.0209 (4)	0.0182 (5)	0.0095 (4)	0.0052 (4)	0.0084 (4)
Mo18	0.0244 (5)	0.0158 (4)	0.0263 (5)	0.0052 (4)	−0.0025 (4)	0.0098 (4)
Mo19	0.0176 (4)	0.0199 (4)	0.0180 (5)	0.0033 (3)	0.0031 (4)	0.0085 (4)
Mo20	0.0152 (4)	0.0223 (4)	0.0170 (4)	0.0061 (3)	0.0030 (3)	0.0055 (4)
Mo21	0.0159 (4)	0.0138 (4)	0.0236 (5)	0.0038 (3)	0.0000 (4)	0.0084 (4)
Mo22	0.0123 (4)	0.0271 (5)	0.0208 (5)	0.0024 (4)	0.0011 (4)	0.0114 (4)
Mo23	0.0212 (5)	0.0215 (4)	0.0168 (5)	0.0071 (4)	−0.0025 (4)	0.0045 (4)
Mo24	0.0199 (4)	0.0137 (4)	0.0203 (5)	0.0017 (3)	−0.0008 (4)	0.0034 (4)
P2	0.0143 (11)	0.0128 (11)	0.0146 (12)	0.0027 (9)	−0.0006 (9)	0.0052 (10)
O41	0.018 (3)	0.024 (4)	0.027 (4)	0.003 (3)	−0.003 (3)	0.013 (3)
O42	0.025 (4)	0.018 (3)	0.023 (4)	0.005 (3)	0.003 (3)	0.008 (3)
O43	0.018 (3)	0.021 (3)	0.021 (4)	0.001 (3)	−0.002 (3)	0.013 (3)
O44	0.020 (3)	0.017 (3)	0.027 (4)	0.008 (3)	0.008 (3)	0.008 (3)
O45	0.027 (4)	0.029 (4)	0.018 (4)	0.008 (3)	0.003 (3)	0.006 (3)
O46	0.039 (4)	0.020 (4)	0.026 (4)	0.004 (3)	0.002 (3)	0.000 (3)
O47	0.015 (3)	0.031 (4)	0.042 (4)	−0.007 (3)	−0.004 (3)	0.020 (3)
O48	0.030 (4)	0.025 (4)	0.023 (4)	0.009 (3)	0.011 (3)	0.015 (3)
O49	0.028 (4)	0.025 (4)	0.022 (4)	0.015 (3)	0.007 (3)	0.009 (3)
O50	0.042 (4)	0.020 (4)	0.027 (4)	0.008 (3)	0.009 (3)	0.009 (3)
O51	0.021 (4)	0.024 (4)	0.032 (4)	0.005 (3)	0.005 (3)	0.009 (3)
O52	0.020 (4)	0.022 (4)	0.032 (4)	0.005 (3)	0.008 (3)	0.003 (3)
O53	0.030 (4)	0.026 (4)	0.025 (4)	0.002 (3)	−0.005 (3)	0.017 (3)
O54	0.029 (4)	0.023 (4)	0.035 (4)	−0.005 (3)	0.001 (3)	0.014 (3)
O55	0.030 (4)	0.015 (3)	0.026 (4)	0.004 (3)	0.004 (3)	0.013 (3)
O56	0.022 (4)	0.025 (4)	0.022 (4)	0.003 (3)	−0.005 (3)	0.009 (3)
O57	0.031 (4)	0.031 (4)	0.020 (4)	0.009 (3)	0.008 (3)	0.013 (3)
O58	0.030 (4)	0.043 (4)	0.022 (4)	0.014 (3)	−0.001 (3)	−0.001 (3)
O59	0.029 (4)	0.029 (4)	0.024 (4)	0.015 (3)	0.009 (3)	0.015 (3)
O60	0.041 (4)	0.026 (4)	0.020 (4)	−0.002 (3)	0.013 (3)	0.010 (3)
O61	0.019 (4)	0.030 (4)	0.026 (4)	0.009 (3)	−0.001 (3)	0.004 (3)
O62	0.022 (4)	0.022 (4)	0.036 (4)	0.004 (3)	−0.002 (3)	0.017 (3)
O63	0.019 (4)	0.021 (3)	0.031 (4)	0.000 (3)	0.008 (3)	0.005 (3)
O64	0.024 (4)	0.024 (3)	0.012 (3)	0.008 (3)	0.006 (3)	0.001 (3)
O65	0.027 (4)	0.026 (4)	0.024 (4)	0.000 (3)	−0.013 (3)	0.011 (3)
O66	0.030 (4)	0.016 (3)	0.035 (4)	0.001 (3)	−0.005 (3)	0.009 (3)
O67	0.027 (4)	0.019 (3)	0.016 (3)	0.007 (3)	−0.002 (3)	0.004 (3)
O68	0.015 (3)	0.042 (4)	0.024 (4)	−0.003 (3)	0.000 (3)	0.012 (3)
O69	0.031 (4)	0.021 (3)	0.023 (4)	0.001 (3)	−0.003 (3)	0.013 (3)
O70	0.023 (4)	0.026 (4)	0.019 (4)	0.005 (3)	−0.002 (3)	0.009 (3)
O71	0.015 (3)	0.025 (4)	0.027 (4)	0.000 (3)	−0.002 (3)	0.006 (3)
O72	0.020 (4)	0.022 (4)	0.033 (4)	0.007 (3)	0.002 (3)	0.006 (3)
O73	0.019 (4)	0.037 (4)	0.023 (4)	0.001 (3)	−0.001 (3)	0.012 (3)
O74	0.022 (4)	0.028 (4)	0.027 (4)	−0.006 (3)	−0.005 (3)	0.013 (3)
O75	0.031 (4)	0.031 (4)	0.024 (4)	0.010 (3)	0.003 (3)	0.011 (3)
O76	0.029 (4)	0.031 (4)	0.033 (4)	0.016 (3)	0.012 (3)	0.019 (3)
O77	0.034 (4)	0.033 (4)	0.017 (4)	0.012 (3)	0.012 (3)	0.010 (3)
O78	0.020 (4)	0.036 (4)	0.032 (4)	0.003 (3)	−0.001 (3)	0.011 (3)
O79	0.020 (4)	0.030 (4)	0.018 (4)	−0.001 (3)	0.001 (3)	0.009 (3)
O80	0.036 (4)	0.009 (3)	0.021 (4)	−0.005 (3)	−0.003 (3)	−0.003 (3)
O1S	0.040 (5)	0.042 (5)	0.045 (5)	0.005 (4)	0.005 (4)	0.018 (4)
O8S	0.068 (6)	0.049 (5)	0.050 (5)	−0.014 (5)	−0.005 (4)	0.034 (4)
N1	0.034 (5)	0.045 (6)	0.033 (6)	0.017 (5)	0.011 (4)	0.010 (5)
N8	0.029 (5)	0.038 (5)	0.061 (7)	0.012 (4)	0.015 (5)	0.029 (5)
C11	0.026 (6)	0.062 (8)	0.038 (7)	0.014 (6)	0.017 (5)	0.023 (7)
C12	0.050 (8)	0.054 (8)	0.044 (8)	0.029 (7)	0.021 (6)	0.023 (6)
C13	0.054 (8)	0.025 (6)	0.022 (6)	0.010 (5)	0.009 (5)	0.002 (5)
C14	0.024 (6)	0.072 (9)	0.024 (6)	0.009 (6)	−0.012 (5)	0.001 (6)
C15	0.061 (9)	0.067 (9)	0.038 (8)	0.034 (7)	0.004 (6)	0.014 (7)
C81	0.045 (7)	0.046 (7)	0.043 (7)	−0.007 (6)	0.009 (6)	0.024 (6)
C82	0.085 (11)	0.070 (10)	0.079 (11)	−0.032 (9)	0.034 (9)	−0.004 (9)
C83	0.18 (2)	0.061 (11)	0.109 (15)	−0.006 (12)	0.048 (14)	−0.018 (10)
C84	0.054 (8)	0.064 (9)	0.053 (9)	0.008 (7)	0.015 (7)	0.032 (7)
C85	0.041 (7)	0.053 (8)	0.088 (10)	0.015 (6)	0.021 (7)	0.045 (8)
O2S	0.107 (8)	0.072 (7)	0.055 (6)	0.008 (6)	0.045 (6)	0.015 (5)
O11S	0.067 (6)	0.041 (5)	0.034 (5)	0.020 (4)	0.019 (4)	0.016 (4)
N2	0.063 (7)	0.085 (8)	0.039 (6)	−0.012 (6)	−0.016 (6)	0.026 (6)
N11	0.044 (5)	0.036 (5)	0.039 (5)	0.000 (4)	0.015 (4)	0.021 (4)
C115	0.074 (10)	0.068 (10)	0.085 (11)	0.002 (8)	0.027 (9)	0.030 (9)
C21	0.069 (8)	0.067 (8)	0.044 (8)	0.013 (7)	0.025 (7)	0.032 (7)
C22	0.057 (8)	0.125 (10)	0.112 (10)	0.035 (8)	0.029 (8)	0.102 (8)
C23	0.077 (12)	0.078 (12)	0.200 (19)	0.032 (10)	−0.013 (12)	0.024 (12)
C24	0.053 (8)	0.107 (11)	0.020 (7)	0.019 (8)	0.011 (6)	−0.007 (7)
C25	0.26 (2)	0.054 (10)	0.064 (11)	−0.019 (13)	−0.045 (13)	0.009 (9)
C111	0.030 (5)	0.036 (5)	0.049 (6)	0.011 (4)	0.017 (5)	0.029 (4)
C112	0.026 (5)	0.035 (6)	0.090 (8)	−0.002 (5)	0.028 (5)	0.032 (5)
C113	0.049 (8)	0.026 (7)	0.151 (14)	0.005 (6)	0.026 (9)	0.031 (8)
C114	0.040 (7)	0.043 (7)	0.053 (8)	0.016 (6)	0.016 (6)	0.008 (6)
O3S	0.032 (4)	0.043 (5)	0.033 (4)	0.005 (4)	0.005 (4)	0.009 (4)
O7S	0.037 (4)	0.023 (4)	0.033 (4)	−0.004 (3)	−0.010 (3)	0.013 (3)
N3	0.025 (5)	0.026 (5)	0.029 (5)	−0.009 (4)	−0.001 (4)	0.006 (4)
N7	0.018 (4)	0.034 (5)	0.021 (5)	0.003 (4)	0.003 (4)	0.005 (4)
C31	0.029 (6)	0.012 (5)	0.044 (7)	−0.006 (4)	0.004 (5)	0.014 (5)
C32	0.032 (6)	0.026 (6)	0.043 (7)	0.017 (5)	−0.002 (5)	0.002 (5)
C33	0.048 (7)	0.026 (6)	0.041 (7)	0.002 (5)	0.011 (6)	0.015 (5)
C34	0.033 (6)	0.034 (6)	0.028 (6)	0.001 (5)	0.005 (5)	0.004 (5)
C35	0.042 (7)	0.038 (7)	0.044 (7)	0.004 (6)	−0.001 (6)	0.019 (6)
C71	0.032 (6)	0.028 (6)	0.022 (6)	0.013 (5)	0.002 (5)	0.011 (5)
C72	0.027 (6)	0.041 (6)	0.022 (6)	0.005 (5)	−0.002 (5)	0.013 (5)
C73	0.035 (7)	0.081 (9)	0.028 (7)	0.022 (7)	0.004 (5)	0.020 (6)
C74	0.021 (5)	0.038 (6)	0.035 (6)	0.006 (5)	0.002 (5)	0.018 (5)
C75	0.022 (5)	0.036 (6)	0.027 (6)	−0.001 (5)	0.002 (5)	0.011 (5)
O4S	0.028 (4)	0.119 (8)	0.037 (5)	0.014 (5)	0.009 (4)	0.049 (5)
O5S	0.028 (4)	0.085 (6)	0.027 (4)	0.009 (4)	−0.001 (3)	0.038 (4)
N4	0.023 (5)	0.052 (6)	0.033 (6)	−0.001 (5)	0.018 (4)	−0.006 (5)
N5	0.033 (5)	0.033 (4)	0.040 (5)	0.020 (4)	0.020 (4)	0.026 (4)
C41	0.019 (6)	0.052 (7)	0.016 (6)	0.001 (5)	0.002 (5)	−0.004 (5)
C42	0.033 (6)	0.015 (5)	0.027 (6)	−0.001 (4)	0.007 (5)	0.006 (4)
C43	0.025 (6)	0.034 (6)	0.018 (5)	0.006 (5)	0.004 (4)	0.008 (5)
C44	0.029 (7)	0.098 (11)	0.022 (7)	0.002 (7)	0.000 (5)	−0.009 (7)
C45	0.024 (7)	0.039 (7)	0.107 (12)	−0.010 (6)	0.030 (7)	−0.021 (8)
C51	0.043 (5)	0.052 (5)	0.039 (5)	0.018 (5)	0.011 (5)	0.028 (5)
C52	0.069 (7)	0.054 (6)	0.037 (6)	0.020 (6)	0.011 (6)	0.021 (5)
C53	0.113 (11)	0.036 (7)	0.036 (7)	0.042 (7)	0.031 (7)	0.019 (6)
C54	0.043 (7)	0.032 (6)	0.035 (7)	0.016 (5)	0.019 (5)	0.016 (5)
C55	0.034 (7)	0.047 (7)	0.049 (8)	−0.003 (6)	0.012 (6)	0.022 (6)
O6S	0.035 (4)	0.026 (4)	0.013 (3)	0.010 (3)	0.001 (3)	0.005 (3)
N6	0.024 (4)	0.032 (4)	0.027 (4)	0.008 (4)	0.006 (4)	0.007 (4)
C61	0.014 (4)	0.038 (5)	0.022 (4)	0.012 (4)	0.012 (4)	0.010 (4)
C62	0.019 (5)	0.036 (5)	0.023 (5)	0.004 (4)	0.001 (4)	0.018 (4)
C63	0.040 (7)	0.032 (6)	0.035 (7)	0.005 (5)	0.002 (5)	0.010 (5)
C64	0.023 (6)	0.054 (7)	0.024 (6)	0.010 (5)	0.003 (5)	0.024 (5)
C65	0.035 (6)	0.043 (7)	0.032 (7)	0.014 (6)	0.000 (5)	−0.002 (5)
O9S	0.044 (5)	0.054 (5)	0.027 (5)	−0.004 (4)	−0.013 (4)	0.001 (4)
N9	0.084 (9)	0.027 (5)	0.048 (7)	0.006 (5)	0.009 (6)	0.024 (5)
C91	0.055 (8)	0.023 (6)	0.034 (7)	−0.009 (5)	−0.008 (6)	0.007 (5)
C92	0.054 (8)	0.034 (7)	0.068 (9)	−0.001 (6)	−0.031 (7)	0.013 (7)
C93	0.037 (7)	0.053 (8)	0.063 (9)	−0.010 (6)	−0.001 (6)	0.024 (7)
C94	0.129 (15)	0.065 (10)	0.090 (12)	0.016 (10)	0.060 (11)	0.050 (9)
C95	0.22 (2)	0.049 (9)	0.027 (8)	0.050 (11)	−0.024 (10)	0.008 (7)
O10S	0.040 (5)	0.052 (5)	0.041 (5)	−0.012 (4)	−0.007 (4)	0.030 (4)
N10	0.044 (6)	0.028 (5)	0.014 (4)	0.007 (4)	0.007 (4)	0.005 (4)
C101	0.034 (6)	0.032 (6)	0.021 (6)	−0.001 (5)	0.013 (5)	0.006 (5)
C102	0.034 (7)	0.026 (6)	0.045 (7)	0.005 (5)	0.001 (6)	0.006 (5)
C103	0.065 (9)	0.040 (8)	0.080 (10)	0.038 (7)	−0.012 (8)	0.009 (7)
C104	0.037 (7)	0.042 (7)	0.027 (6)	0.008 (5)	−0.001 (5)	0.005 (5)
C105	0.063 (9)	0.036 (7)	0.051 (8)	0.006 (6)	0.034 (7)	0.017 (6)

### Geometric parameters (Å, º)

**Table:**

Mo1—O1	2.425 (5)	C81—C82	1.483 (15)
Mo1—O5	1.672 (5)	C82—H82A	0.9900
Mo1—O8	1.888 (5)	C82—H82B	0.9900
Mo1—O9	1.996 (5)	C82—C83	1.499 (9)
Mo1—O11	1.818 (5)	C83—H83A	0.9800
Mo1—O12	1.976 (6)	C83—H83B	0.9800
Mo2—O1	2.433 (5)	C83—H83C	0.9800
Mo2—O6	1.684 (5)	C84—H84A	0.9800
Mo2—O9	1.841 (5)	C84—H84B	0.9800
Mo2—O10	2.012 (5)	C84—H84C	0.9800
Mo2—O13	1.820 (5)	C85—H85A	0.9800
Mo2—O14	2.013 (6)	C85—H85B	0.9800
Mo3—O1	2.410 (5)	C85—H85C	0.9800
Mo3—O7	1.677 (6)	O2S—H2S	1.22
Mo3—O8	2.033 (5)	O2S—C21	1.237 (14)
Mo3—O10	1.834 (5)	O11S—H2S	1.21
Mo3—O15	1.814 (5)	O11S—C111	1.242 (11)
Mo3—O16	1.992 (5)	N2—C21	1.316 (15)
Mo4—O2	2.450 (5)	N2—C24	1.550 (14)
Mo4—O12	1.852 (6)	N2—C25	1.480 (16)
Mo4—O17	1.664 (5)	N11—C115	1.418 (13)
Mo4—O23	2.005 (5)	N11—C111	1.373 (12)
Mo4—O24	1.820 (6)	N11—C114	1.467 (12)
Mo4—O29	2.011 (5)	C115—H11A	0.9800
Mo5—O2	2.409 (5)	C115—H11B	0.9800
Mo5—O13	2.026 (5)	C115—H11C	0.9800
Mo5—O18	1.674 (6)	C21—C22	1.452 (9)
Mo5—O24	2.005 (6)	C22—H22A	0.9900
Mo5—O25	1.821 (6)	C22—H22B	0.9900
Mo5—O30	1.833 (5)	C22—C23	1.469 (9)
Mo6—O3	2.439 (5)	C23—H23A	0.9800
Mo6—O14	1.824 (5)	C23—H23B	0.9800
Mo6—O19	1.669 (6)	C23—H23C	0.9800
Mo6—O25	2.004 (6)	C24—H24A	0.9800
Mo6—O26	1.814 (6)	C24—H24B	0.9800
Mo6—O31	2.009 (6)	C24—H24C	0.9800
Mo7—O3	2.411 (5)	C25—H25A	0.9800
Mo7—O15	2.021 (5)	C25—H25B	0.9800
Mo7—O20	1.667 (6)	C25—H25C	0.9800
Mo7—O26	2.031 (6)	C111—C112	1.483 (13)
Mo7—O27	1.814 (6)	C112—H11D	0.9900
Mo7—O32	1.832 (6)	C112—H11E	0.9900
Mo8—O4	2.422 (5)	C112—C113	1.459 (14)
Mo8—O16	1.834 (5)	C113—H11F	0.9800
Mo8—O21	1.670 (6)	C113—H11G	0.9800
Mo8—O27	2.024 (6)	C113—H11H	0.9800
Mo8—O28	1.844 (6)	C114—H11I	0.9800
Mo8—O33	1.988 (6)	C114—H11J	0.9800
Mo9—O4	2.440 (5)	C114—H11K	0.9800
Mo9—O11	2.028 (5)	O3S—H3S	1.21
Mo9—O22	1.670 (5)	O3S—C31	1.271 (11)
Mo9—O23	1.831 (6)	O7S—H3S	1.22
Mo9—O28	1.993 (6)	O7S—C71	1.271 (10)
Mo9—O34	1.824 (5)	N3—C31	1.322 (11)
Mo10—O2	2.435 (5)	N3—C34	1.434 (11)
Mo10—O29	1.833 (6)	N3—C35	1.475 (11)
Mo10—O30	2.011 (6)	N7—C71	1.298 (11)
Mo10—O35	1.816 (5)	N7—C74	1.461 (11)
Mo10—O36	2.005 (6)	N7—C75	1.430 (11)
Mo10—O38	1.676 (5)	C31—C32	1.520 (13)
Mo11—O3	2.441 (5)	C32—H32A	0.9900
Mo11—O31	1.827 (6)	C32—H32B	0.9900
Mo11—O32	1.996 (6)	C32—C33	1.521 (12)
Mo11—O36	1.823 (6)	C33—H33A	0.9800
Mo11—O37	2.042 (6)	C33—H33B	0.9800
Mo11—O39	1.679 (6)	C33—H33C	0.9800
Mo12—O4	2.427 (5)	C34—H34A	0.9800
Mo12—O33	1.850 (6)	C34—H34B	0.9800
Mo12—O34	2.030 (6)	C34—H34C	0.9800
Mo12—O35	2.010 (5)	C35—H35A	0.9800
Mo12—O37	1.808 (5)	C35—H35B	0.9800
Mo12—O40	1.667 (5)	C35—H35C	0.9800
P1—O1	1.541 (5)	C71—C72	1.514 (12)
P1—O2	1.536 (6)	C72—H72A	0.9900
P1—O3	1.546 (6)	C72—H72B	0.9900
P1—O4	1.533 (5)	C72—C73	1.519 (12)
Mo13—O41	2.439 (6)	C73—H73A	0.9800
Mo13—O45	1.674 (6)	C73—H73B	0.9800
Mo13—O48	1.997 (6)	C73—H73C	0.9800
Mo13—O49	1.832 (6)	C74—H74A	0.9800
Mo13—O51	2.014 (6)	C74—H74B	0.9800
Mo13—O52	1.811 (6)	C74—H74C	0.9800
Mo14—O41	2.454 (6)	C75—H75A	0.9800
Mo14—O46	1.656 (6)	C75—H75B	0.9800
Mo14—O49	2.010 (6)	C75—H75C	0.9800
Mo14—O50	1.842 (6)	O4S—H4S	1.22
Mo14—O53	1.986 (6)	O4S—C41	1.241 (12)
Mo14—O54	1.821 (6)	O5S—H4S	1.23
Mo15—O41	2.408 (6)	O5S—C51	1.284 (11)
Mo15—O47	1.662 (6)	N4—C41	1.309 (12)
Mo15—O48	1.848 (6)	N4—C44	1.462 (13)
Mo15—O50	2.023 (6)	N4—C45	1.460 (14)
Mo15—O55	1.998 (6)	N5—C51	1.270 (12)
Mo15—O56	1.815 (6)	N5—C54	1.475 (11)
Mo16—O42	2.460 (6)	N5—C55	1.451 (12)
Mo16—O52	1.988 (6)	C41—C42	1.516 (13)
Mo16—O57	1.678 (6)	C42—H42A	0.9900
Mo16—O63	1.799 (6)	C42—H42B	0.9900
Mo16—O64	2.007 (6)	C42—C43	1.484 (11)
Mo16—O69	1.834 (6)	C43—H43A	0.9800
Mo17—O42	2.452 (6)	C43—H43B	0.9800
Mo17—O53	1.836 (6)	C43—H43C	0.9800
Mo17—O58	1.660 (6)	C44—H44A	0.9800
Mo17—O64	1.835 (6)	C44—H44B	0.9800
Mo17—O65	2.017 (6)	C44—H44C	0.9800
Mo17—O70	1.990 (6)	C45—H45A	0.9800
Mo18—O43	2.446 (5)	C45—H45B	0.9800
Mo18—O54	2.002 (6)	C45—H45C	0.9800
Mo18—O59	1.665 (6)	C51—C52	1.569 (15)
Mo18—O65	1.800 (6)	C52—H52A	0.9900
Mo18—O66	2.031 (6)	C52—H52B	0.9900
Mo18—O71	1.845 (6)	C52—C53	1.438 (14)
Mo19—O43	2.423 (6)	C53—H53A	0.9800
Mo19—O55	1.822 (6)	C53—H53B	0.9800
Mo19—O60	1.683 (6)	C53—H53C	0.9800
Mo19—O66	1.840 (6)	C54—H54A	0.9800
Mo19—O67	2.001 (6)	C54—H54B	0.9800
Mo19—O72	1.996 (6)	C54—H54C	0.9800
Mo20—O44	2.431 (6)	C55—H55A	0.9800
Mo20—O56	2.002 (6)	C55—H55B	0.9800
Mo20—O61	1.659 (6)	C55—H55C	0.9800
Mo20—O67	1.816 (6)	O6S—H6S	0.8400
Mo20—O68	2.003 (6)	O6S—C61	1.309 (10)
Mo20—O73	1.830 (6)	N6—C61	1.296 (11)
Mo21—O44	2.436 (6)	N6—C64	1.493 (11)
Mo21—O51	1.816 (6)	N6—C65	1.462 (11)
Mo21—O62	1.666 (6)	C61—C62	1.467 (12)
Mo21—O63	2.005 (6)	C62—H62A	0.9900
Mo21—O68	1.834 (6)	C62—H62B	0.9900
Mo21—O74	2.014 (6)	C62—C63	1.541 (12)
Mo22—O42	2.442 (6)	C63—H63A	0.9800
Mo22—O69	2.025 (6)	C63—H63B	0.9800
Mo22—O70	1.839 (6)	C63—H63C	0.9800
Mo22—O75	1.998 (6)	C64—H64A	0.9800
Mo22—O76	1.837 (6)	C64—H64B	0.9800
Mo22—O78	1.660 (6)	C64—H64C	0.9800
Mo23—O43	2.429 (6)	C65—H65A	0.9800
Mo23—O71	2.013 (6)	C65—H65B	0.9800
Mo23—O72	1.856 (6)	C65—H65C	0.9800
Mo23—O76	1.979 (6)	O9S—H9S	1.22
Mo23—O77	1.815 (6)	O9S—C91	1.256 (11)
Mo23—O79	1.669 (6)	N9—C91	1.295 (13)
Mo24—O44	2.447 (6)	N9—C94	1.455 (15)
Mo24—O73	2.009 (6)	N9—C95	1.484 (14)
Mo24—O74	1.833 (6)	C91—C92	1.513 (15)
Mo24—O75	1.816 (6)	C92—H92A	0.9900
Mo24—O77	2.017 (6)	C92—H92B	0.9900
Mo24—O80	1.663 (5)	C92—C93	1.473 (13)
P2—O41	1.541 (6)	C93—H93A	0.9800
P2—O42	1.507 (6)	C93—H93B	0.9800
P2—O43	1.537 (6)	C93—H93C	0.9800
P2—O44	1.539 (6)	C94—H94A	0.9800
O1S—H1S	1.22	C94—H94B	0.9800
O1S—C11	1.275 (12)	C94—H94C	0.9800
O8S—H1S	1.22	C95—H95A	0.9800
O8S—C81	1.251 (12)	C95—H95B	0.9800
N1—C11	1.308 (12)	C95—H95C	0.9800
N1—C14	1.452 (11)	O10S—H10S	1.22
N1—C15	1.489 (12)	O10S—C101	1.268 (11)
N8—C81	1.303 (12)	N10—C101	1.323 (12)
N8—C84	1.460 (13)	N10—C104	1.478 (11)
N8—C85	1.477 (12)	N10—C105	1.434 (11)
C11—C12	1.483 (14)	C101—C102	1.457 (13)
C12—H12A	0.9900	C102—H10A	0.9900
C12—H12B	0.9900	C102—H10B	0.9900
C12—C13	1.529 (13)	C102—C103	1.490 (13)
C13—H13A	0.9800	C103—H10C	0.9800
C13—H13B	0.9800	C103—H10D	0.9800
C13—H13C	0.9800	C103—H10E	0.9800
C14—H14A	0.9800	C104—H10F	0.9800
C14—H14B	0.9800	C104—H10G	0.9800
C14—H14C	0.9800	C104—H10H	0.9800
C15—H15A	0.9800	C105—H10I	0.9800
C15—H15B	0.9800	C105—H10J	0.9800
C15—H15C	0.9800	C105—H10K	0.9800
			
O5—Mo1—O1	170.1 (2)	O75—Mo24—O73	154.5 (3)
O5—Mo1—O8	101.8 (3)	O75—Mo24—O74	98.1 (3)
O5—Mo1—O9	98.8 (2)	O75—Mo24—O77	85.0 (2)
O5—Mo1—O11	103.7 (2)	O77—Mo24—O44	81.2 (2)
O5—Mo1—O12	101.4 (3)	O80—Mo24—O44	168.1 (3)
O8—Mo1—O1	74.0 (2)	O80—Mo24—O73	98.8 (3)
O8—Mo1—O9	86.3 (2)	O80—Mo24—O74	102.5 (3)
O8—Mo1—O12	155.2 (2)	O80—Mo24—O75	104.6 (3)
O9—Mo1—O1	72.21 (19)	O80—Mo24—O77	101.4 (3)
O11—Mo1—O1	85.8 (2)	O42—P2—O41	110.6 (3)
O11—Mo1—O8	96.9 (2)	O42—P2—O43	109.5 (3)
O11—Mo1—O9	156.1 (2)	O42—P2—O44	110.4 (3)
O11—Mo1—O12	86.2 (2)	O43—P2—O41	108.6 (3)
O12—Mo1—O1	81.72 (19)	O43—P2—O44	109.1 (3)
O12—Mo1—O9	81.5 (2)	O44—P2—O41	108.6 (3)
O6—Mo2—O1	170.9 (2)	Mo13—O41—Mo14	88.84 (19)
O6—Mo2—O9	102.8 (3)	Mo15—O41—Mo13	90.10 (19)
O6—Mo2—O10	100.0 (2)	Mo15—O41—Mo14	90.19 (19)
O6—Mo2—O13	102.9 (3)	P2—O41—Mo13	124.8 (3)
O6—Mo2—O14	100.8 (3)	P2—O41—Mo14	125.1 (3)
O9—Mo2—O1	74.4 (2)	P2—O41—Mo15	126.5 (3)
O9—Mo2—O10	86.2 (2)	Mo17—O42—Mo16	89.16 (19)
O9—Mo2—O14	154.7 (2)	Mo22—O42—Mo16	89.00 (19)
O10—Mo2—O1	71.34 (19)	Mo22—O42—Mo17	89.14 (19)
O10—Mo2—O14	80.7 (2)	P2—O42—Mo16	125.3 (3)
O13—Mo2—O1	86.2 (2)	P2—O42—Mo17	126.1 (3)
O13—Mo2—O9	98.1 (2)	P2—O42—Mo22	126.2 (3)
O13—Mo2—O10	155.2 (2)	Mo19—O43—Mo18	89.62 (18)
O13—Mo2—O14	85.6 (2)	Mo19—O43—Mo23	89.88 (19)
O14—Mo2—O1	80.9 (2)	Mo23—O43—Mo18	89.56 (18)
O7—Mo3—O1	169.6 (2)	P2—O43—Mo18	125.4 (3)
O7—Mo3—O8	97.8 (2)	P2—O43—Mo19	125.9 (3)
O7—Mo3—O10	103.0 (3)	P2—O43—Mo23	125.2 (3)
O7—Mo3—O15	103.0 (3)	Mo20—O44—Mo21	90.00 (18)
O7—Mo3—O16	100.7 (3)	Mo20—O44—Mo24	89.67 (19)
O8—Mo3—O1	72.04 (19)	Mo21—O44—Mo24	89.30 (18)
O10—Mo3—O1	74.7 (2)	P2—O44—Mo20	126.5 (3)
O10—Mo3—O8	86.0 (2)	P2—O44—Mo21	125.0 (3)
O10—Mo3—O16	154.1 (2)	P2—O44—Mo24	125.0 (3)
O15—Mo3—O1	87.4 (2)	Mo15—O48—Mo13	126.3 (3)
O15—Mo3—O8	156.9 (2)	Mo13—O49—Mo14	126.0 (3)
O15—Mo3—O10	99.0 (2)	Mo14—O50—Mo15	125.9 (3)
O15—Mo3—O16	85.6 (2)	Mo21—O51—Mo13	150.6 (3)
O16—Mo3—O1	80.1 (2)	Mo13—O52—Mo16	152.3 (3)
O16—Mo3—O8	80.6 (2)	Mo17—O53—Mo14	152.1 (3)
O12—Mo4—O2	86.2 (2)	Mo14—O54—Mo18	151.2 (3)
O12—Mo4—O23	85.2 (2)	Mo19—O55—Mo15	149.3 (3)
O12—Mo4—O29	155.0 (2)	Mo15—O56—Mo20	150.4 (3)
O17—Mo4—O2	170.5 (2)	Mo16—O63—Mo21	151.4 (3)
O17—Mo4—O12	103.1 (3)	Mo17—O64—Mo16	127.6 (3)
O17—Mo4—O23	101.7 (3)	Mo18—O65—Mo17	150.8 (3)
O17—Mo4—O24	102.9 (3)	Mo19—O66—Mo18	124.8 (3)
O17—Mo4—O29	99.9 (3)	Mo20—O67—Mo19	151.9 (3)
O23—Mo4—O2	80.95 (19)	Mo21—O68—Mo20	127.5 (3)
O23—Mo4—O29	80.8 (2)	Mo16—O69—Mo22	125.8 (3)
O24—Mo4—O2	73.5 (2)	Mo22—O70—Mo17	127.5 (3)
O24—Mo4—O12	97.2 (2)	Mo18—O71—Mo23	125.7 (3)
O24—Mo4—O23	154.1 (2)	Mo23—O72—Mo19	125.6 (3)
O24—Mo4—O29	87.0 (2)	Mo20—O73—Mo24	127.2 (3)
O29—Mo4—O2	71.3 (2)	Mo24—O74—Mo21	126.2 (3)
O13—Mo5—O2	80.74 (19)	Mo24—O75—Mo22	151.6 (4)
O18—Mo5—O2	170.7 (3)	Mo22—O76—Mo23	151.3 (3)
O18—Mo5—O13	100.8 (2)	Mo23—O77—Mo24	150.7 (3)
O18—Mo5—O24	99.5 (3)	C11—O1S—H1S	111
O18—Mo5—O25	103.0 (3)	C81—O8S—H1S	130
O18—Mo5—O30	102.4 (3)	C11—N1—C14	123.5 (10)
O24—Mo5—O2	71.7 (2)	C11—N1—C15	123.0 (10)
O24—Mo5—O13	79.9 (2)	C14—N1—C15	113.5 (9)
O25—Mo5—O2	86.3 (2)	C81—N8—C84	120.2 (9)
O25—Mo5—O13	84.6 (2)	C81—N8—C85	124.4 (10)
O25—Mo5—O24	154.7 (2)	C84—N8—C85	115.2 (9)
O25—Mo5—O30	99.0 (3)	O1S—C11—N1	117.1 (11)
O30—Mo5—O2	74.9 (2)	O1S—C11—C12	121.0 (10)
O30—Mo5—O13	155.0 (2)	N1—C11—C12	121.8 (11)
O30—Mo5—O24	87.2 (2)	C11—C12—H12A	109.5
O14—Mo6—O3	85.6 (2)	C11—C12—H12B	109.5
O14—Mo6—O25	84.7 (2)	C11—C12—C13	110.8 (8)
O14—Mo6—O31	154.1 (2)	H12A—C12—H12B	108.1
O19—Mo6—O3	171.2 (3)	C13—C12—H12A	109.5
O19—Mo6—O14	103.2 (3)	C13—C12—H12B	109.5
O19—Mo6—O25	100.9 (3)	C12—C13—H13A	109.5
O19—Mo6—O26	103.7 (3)	C12—C13—H13B	109.5
O19—Mo6—O31	100.3 (3)	C12—C13—H13C	109.5
O25—Mo6—O3	80.4 (2)	H13A—C13—H13B	109.5
O25—Mo6—O31	80.3 (2)	H13A—C13—H13C	109.5
O26—Mo6—O3	74.0 (2)	H13B—C13—H13C	109.5
O26—Mo6—O14	98.3 (3)	N1—C14—H14A	109.5
O26—Mo6—O25	153.9 (2)	N1—C14—H14B	109.5
O26—Mo6—O31	86.6 (2)	N1—C14—H14C	109.5
O31—Mo6—O3	71.2 (2)	H14A—C14—H14B	109.5
O15—Mo7—O3	81.0 (2)	H14A—C14—H14C	109.5
O15—Mo7—O26	80.2 (2)	H14B—C14—H14C	109.5
O20—Mo7—O3	169.4 (3)	N1—C15—H15A	109.5
O20—Mo7—O15	99.8 (3)	N1—C15—H15B	109.5
O20—Mo7—O26	98.3 (3)	N1—C15—H15C	109.5
O20—Mo7—O27	104.0 (3)	H15A—C15—H15B	109.5
O20—Mo7—O32	103.1 (3)	H15A—C15—H15C	109.5
O26—Mo7—O3	71.3 (2)	H15B—C15—H15C	109.5
O27—Mo7—O3	86.6 (2)	O8S—C81—N8	118.9 (11)
O27—Mo7—O15	85.2 (2)	O8S—C81—C82	120.8 (11)
O27—Mo7—O26	155.1 (2)	N8—C81—C82	120.2 (11)
O27—Mo7—O32	98.5 (3)	C81—C82—H82A	108.1
O32—Mo7—O3	74.7 (2)	C81—C82—H82B	108.1
O32—Mo7—O15	155.1 (2)	C81—C82—C83	117.0 (11)
O32—Mo7—O26	86.9 (2)	H82A—C82—H82B	107.3
O16—Mo8—O4	85.2 (2)	C83—C82—H82A	108.1
O16—Mo8—O27	83.7 (2)	C83—C82—H82B	108.1
O16—Mo8—O28	97.5 (2)	C82—C83—H83A	109.5
O16—Mo8—O33	154.1 (2)	C82—C83—H83B	109.5
O21—Mo8—O4	171.4 (2)	C82—C83—H83C	109.5
O21—Mo8—O16	103.3 (3)	H83A—C83—H83B	109.5
O21—Mo8—O27	101.5 (3)	H83A—C83—H83C	109.5
O21—Mo8—O28	103.0 (3)	H83B—C83—H83C	109.5
O21—Mo8—O33	100.3 (3)	N8—C84—H84A	109.5
O27—Mo8—O4	80.6 (2)	N8—C84—H84B	109.5
O28—Mo8—O4	74.2 (2)	N8—C84—H84C	109.5
O28—Mo8—O27	154.5 (2)	H84A—C84—H84B	109.5
O28—Mo8—O33	87.3 (2)	H84A—C84—H84C	109.5
O33—Mo8—O4	71.7 (2)	H84B—C84—H84C	109.5
O33—Mo8—O27	81.3 (2)	N8—C85—H85A	109.5
O11—Mo9—O4	80.31 (19)	N8—C85—H85B	109.5
O22—Mo9—O4	170.8 (3)	N8—C85—H85C	109.5
O22—Mo9—O11	101.0 (2)	H85A—C85—H85B	109.5
O22—Mo9—O23	103.3 (3)	H85A—C85—H85C	109.5
O22—Mo9—O28	99.7 (3)	H85B—C85—H85C	109.5
O22—Mo9—O34	103.4 (3)	C21—O2S—H2S	118
O23—Mo9—O4	85.8 (2)	C111—O11S—H2S	129
O23—Mo9—O11	84.2 (2)	C21—N2—C24	116.0 (10)
O23—Mo9—O28	154.1 (2)	C21—N2—C25	114.8 (13)
O28—Mo9—O4	71.4 (2)	C25—N2—C24	129.3 (13)
O28—Mo9—O11	80.0 (2)	C115—N11—C114	115.7 (9)
O34—Mo9—O4	74.3 (2)	C111—N11—C115	124.6 (10)
O34—Mo9—O11	154.3 (2)	C111—N11—C114	119.6 (9)
O34—Mo9—O23	97.8 (2)	N11—C115—H11A	109.5
O34—Mo9—O28	88.0 (2)	N11—C115—H11B	109.5
O29—Mo10—O2	74.4 (2)	N11—C115—H11C	109.5
O29—Mo10—O30	87.2 (2)	H11A—C115—H11B	109.5
O29—Mo10—O36	154.2 (2)	H11A—C115—H11C	109.5
O30—Mo10—O2	71.5 (2)	H11B—C115—H11C	109.5
O35—Mo10—O2	86.4 (2)	O2S—C21—N2	118.4 (11)
O35—Mo10—O29	97.2 (2)	O2S—C21—C22	122.0 (14)
O35—Mo10—O30	155.4 (2)	N2—C21—C22	119.5 (14)
O35—Mo10—O36	85.5 (2)	C21—C22—H22A	107.6
O36—Mo10—O2	80.2 (2)	C21—C22—H22B	107.6
O36—Mo10—O30	80.4 (2)	C21—C22—C23	118.7 (11)
O38—Mo10—O2	170.2 (2)	H22A—C22—H22B	107.1
O38—Mo10—O29	103.5 (3)	C23—C22—H22A	107.6
O38—Mo10—O30	99.0 (3)	C23—C22—H22B	107.6
O38—Mo10—O35	103.4 (3)	C22—C23—H23A	109.5
O38—Mo10—O36	100.8 (3)	C22—C23—H23B	109.5
O31—Mo11—O3	73.9 (2)	C22—C23—H23C	109.5
O31—Mo11—O32	87.8 (3)	H23A—C23—H23B	109.5
O31—Mo11—O37	154.1 (2)	H23A—C23—H23C	109.5
O32—Mo11—O3	71.4 (2)	H23B—C23—H23C	109.5
O32—Mo11—O37	80.0 (2)	N2—C24—H24A	109.5
O36—Mo11—O3	86.1 (2)	N2—C24—H24B	109.5
O36—Mo11—O31	98.1 (3)	N2—C24—H24C	109.5
O36—Mo11—O32	154.2 (2)	H24A—C24—H24B	109.5
O36—Mo11—O37	84.2 (2)	H24A—C24—H24C	109.5
O37—Mo11—O3	80.5 (2)	H24B—C24—H24C	109.5
O39—Mo11—O3	170.4 (3)	N2—C25—H25A	109.5
O39—Mo11—O31	104.0 (3)	N2—C25—H25B	109.5
O39—Mo11—O32	99.3 (3)	N2—C25—H25C	109.5
O39—Mo11—O36	103.5 (3)	H25A—C25—H25B	109.5
O39—Mo11—O37	100.5 (3)	H25A—C25—H25C	109.5
O33—Mo12—O4	73.7 (2)	H25B—C25—H25C	109.5
O33—Mo12—O34	85.8 (2)	O11S—C111—N11	115.4 (9)
O33—Mo12—O35	154.5 (2)	O11S—C111—C112	127.5 (10)
O34—Mo12—O4	71.45 (19)	N11—C111—C112	116.9 (10)
O35—Mo12—O4	81.57 (19)	C111—C112—H11D	109.0
O35—Mo12—O34	80.5 (2)	C111—C112—H11E	109.0
O37—Mo12—O4	86.9 (2)	H11D—C112—H11E	107.8
O37—Mo12—O33	98.7 (3)	C113—C112—C111	113.0 (11)
O37—Mo12—O34	155.8 (2)	C113—C112—H11D	109.0
O37—Mo12—O35	85.8 (2)	C113—C112—H11E	109.0
O40—Mo12—O4	168.4 (2)	C112—C113—H11F	109.5
O40—Mo12—O33	102.5 (3)	C112—C113—H11G	109.5
O40—Mo12—O34	97.6 (2)	C112—C113—H11H	109.5
O40—Mo12—O35	100.6 (2)	H11F—C113—H11G	109.5
O40—Mo12—O37	104.5 (3)	H11F—C113—H11H	109.5
O1—P1—O3	109.3 (3)	H11G—C113—H11H	109.5
O2—P1—O1	110.2 (3)	N11—C114—H11I	109.5
O2—P1—O3	108.4 (3)	N11—C114—H11J	109.5
O4—P1—O1	109.3 (3)	N11—C114—H11K	109.5
O4—P1—O2	110.0 (3)	H11I—C114—H11J	109.5
O4—P1—O3	109.7 (3)	H11I—C114—H11K	109.5
Mo1—O1—Mo2	88.77 (17)	H11J—C114—H11K	109.5
Mo3—O1—Mo1	90.90 (17)	C31—O3S—H3S	115
Mo3—O1—Mo2	89.38 (17)	C71—O7S—H3S	116
P1—O1—Mo1	125.6 (3)	C31—N3—C34	121.9 (9)
P1—O1—Mo2	125.3 (3)	C31—N3—C35	124.1 (9)
P1—O1—Mo3	125.6 (3)	C34—N3—C35	114.0 (8)
Mo5—O2—Mo4	89.02 (17)	C71—N7—C74	119.6 (8)
Mo5—O2—Mo10	89.20 (17)	C71—N7—C75	125.3 (8)
Mo10—O2—Mo4	88.81 (18)	C75—N7—C74	115.1 (8)
P1—O2—Mo4	124.9 (3)	O3S—C31—N3	118.5 (9)
P1—O2—Mo5	126.7 (3)	O3S—C31—C32	118.6 (9)
P1—O2—Mo10	126.3 (3)	N3—C31—C32	122.9 (9)
Mo6—O3—Mo11	88.89 (18)	C31—C32—H32A	109.4
Mo7—O3—Mo6	89.51 (18)	C31—C32—H32B	109.4
Mo7—O3—Mo11	88.87 (17)	C31—C32—C33	111.2 (8)
P1—O3—Mo6	126.1 (3)	H32A—C32—H32B	108.0
P1—O3—Mo7	125.9 (3)	C33—C32—H32A	109.4
P1—O3—Mo11	125.8 (3)	C33—C32—H32B	109.4
Mo8—O4—Mo9	89.04 (17)	C32—C33—H33A	109.5
Mo8—O4—Mo12	89.36 (17)	C32—C33—H33B	109.5
Mo12—O4—Mo9	89.25 (17)	C32—C33—H33C	109.5
P1—O4—Mo8	126.1 (3)	H33A—C33—H33B	109.5
P1—O4—Mo9	125.8 (3)	H33A—C33—H33C	109.5
P1—O4—Mo12	125.5 (3)	H33B—C33—H33C	109.5
Mo1—O8—Mo3	123.0 (3)	N3—C34—H34A	109.5
Mo2—O9—Mo1	124.6 (3)	N3—C34—H34B	109.5
Mo3—O10—Mo2	124.6 (3)	N3—C34—H34C	109.5
Mo1—O11—Mo9	151.8 (3)	H34A—C34—H34B	109.5
Mo4—O12—Mo1	151.0 (3)	H34A—C34—H34C	109.5
Mo2—O13—Mo5	151.2 (3)	H34B—C34—H34C	109.5
Mo6—O14—Mo2	151.9 (3)	N3—C35—H35A	109.5
Mo3—O15—Mo7	150.4 (3)	N3—C35—H35B	109.5
Mo8—O16—Mo3	153.1 (3)	N3—C35—H35C	109.5
Mo9—O23—Mo4	152.7 (3)	H35A—C35—H35B	109.5
Mo4—O24—Mo5	125.8 (3)	H35A—C35—H35C	109.5
Mo5—O25—Mo6	153.0 (3)	H35B—C35—H35C	109.5
Mo6—O26—Mo7	125.2 (3)	O7S—C71—N7	120.2 (9)
Mo7—O27—Mo8	152.4 (3)	O7S—C71—C72	118.9 (9)
Mo8—O28—Mo9	125.2 (3)	N7—C71—C72	120.7 (9)
Mo10—O29—Mo4	125.5 (3)	C71—C72—H72A	109.5
Mo5—O30—Mo10	124.4 (3)	C71—C72—H72B	109.5
Mo11—O31—Mo6	125.9 (3)	C71—C72—C73	110.8 (8)
Mo7—O32—Mo11	125.0 (3)	H72A—C72—H72B	108.1
Mo12—O33—Mo8	125.3 (3)	C73—C72—H72A	109.5
Mo9—O34—Mo12	124.9 (3)	C73—C72—H72B	109.5
Mo10—O35—Mo12	150.9 (3)	C72—C73—H73A	109.5
Mo11—O36—Mo10	152.7 (3)	C72—C73—H73B	109.5
Mo12—O37—Mo11	151.3 (3)	C72—C73—H73C	109.5
O45—Mo13—O41	168.9 (3)	H73A—C73—H73B	109.5
O45—Mo13—O48	99.4 (3)	H73A—C73—H73C	109.5
O45—Mo13—O49	102.0 (3)	H73B—C73—H73C	109.5
O45—Mo13—O51	101.3 (3)	N7—C74—H74A	109.5
O45—Mo13—O52	104.4 (3)	N7—C74—H74B	109.5
O48—Mo13—O41	70.3 (2)	N7—C74—H74C	109.5
O48—Mo13—O51	80.5 (2)	H74A—C74—H74B	109.5
O49—Mo13—O41	74.1 (2)	H74A—C74—H74C	109.5
O49—Mo13—O48	87.5 (2)	H74B—C74—H74C	109.5
O49—Mo13—O51	155.3 (2)	N7—C75—H75A	109.5
O51—Mo13—O41	81.5 (2)	N7—C75—H75B	109.5
O52—Mo13—O41	86.5 (2)	N7—C75—H75C	109.5
O52—Mo13—O48	154.1 (3)	H75A—C75—H75B	109.5
O52—Mo13—O49	97.3 (3)	H75A—C75—H75C	109.5
O52—Mo13—O51	85.0 (2)	H75B—C75—H75C	109.5
O46—Mo14—O41	169.3 (3)	C41—O4S—H4S	128
O46—Mo14—O49	99.4 (3)	C51—O5S—H4S	112
O46—Mo14—O50	102.6 (3)	C41—N4—C44	120.3 (10)
O46—Mo14—O53	101.9 (3)	C41—N4—C45	123.5 (10)
O46—Mo14—O54	103.6 (3)	C45—N4—C44	116.2 (9)
O49—Mo14—O41	71.0 (2)	C51—N5—C54	123.9 (9)
O50—Mo14—O41	72.8 (2)	C51—N5—C55	119.0 (9)
O50—Mo14—O49	87.1 (2)	C55—N5—C54	116.5 (8)
O50—Mo14—O53	153.9 (3)	O4S—C41—N4	119.1 (10)
O53—Mo14—O41	81.5 (2)	O4S—C41—C42	121.3 (9)
O53—Mo14—O49	80.2 (2)	N4—C41—C42	119.6 (10)
O54—Mo14—O41	86.8 (2)	C41—C42—H42A	108.9
O54—Mo14—O49	154.7 (3)	C41—C42—H42B	108.9
O54—Mo14—O50	98.1 (3)	H42A—C42—H42B	107.7
O54—Mo14—O53	84.8 (2)	C43—C42—C41	113.6 (8)
O47—Mo15—O41	168.6 (3)	C43—C42—H42A	108.9
O47—Mo15—O48	102.0 (3)	C43—C42—H42B	108.9
O47—Mo15—O50	98.5 (3)	C42—C43—H43A	109.5
O47—Mo15—O55	101.0 (3)	C42—C43—H43B	109.5
O47—Mo15—O56	103.3 (3)	C42—C43—H43C	109.5
O48—Mo15—O41	73.3 (2)	H43A—C43—H43B	109.5
O48—Mo15—O50	85.8 (2)	H43A—C43—H43C	109.5
O48—Mo15—O55	154.8 (3)	H43B—C43—H43C	109.5
O50—Mo15—O41	71.1 (2)	N4—C44—H44A	109.5
O55—Mo15—O41	82.2 (2)	N4—C44—H44B	109.5
O55—Mo15—O50	80.6 (2)	N4—C44—H44C	109.5
O56—Mo15—O41	87.7 (2)	H44A—C44—H44B	109.5
O56—Mo15—O48	98.4 (3)	H44A—C44—H44C	109.5
O56—Mo15—O50	156.3 (3)	H44B—C44—H44C	109.5
O56—Mo15—O55	86.3 (2)	N4—C45—H45A	109.5
O52—Mo16—O42	80.4 (2)	N4—C45—H45B	109.5
O52—Mo16—O64	80.3 (2)	N4—C45—H45C	109.5
O57—Mo16—O42	168.7 (2)	H45A—C45—H45B	109.5
O57—Mo16—O52	102.0 (3)	H45A—C45—H45C	109.5
O57—Mo16—O63	104.4 (3)	H45B—C45—H45C	109.5
O57—Mo16—O64	99.1 (3)	O5S—C51—C52	117.6 (9)
O57—Mo16—O69	102.4 (3)	N5—C51—O5S	119.8 (10)
O63—Mo16—O42	86.7 (2)	N5—C51—C52	122.3 (10)
O63—Mo16—O52	85.5 (2)	C51—C52—H52A	110.6
O63—Mo16—O64	154.6 (3)	C51—C52—H52B	110.6
O63—Mo16—O69	97.7 (3)	H52A—C52—H52B	108.8
O64—Mo16—O42	70.3 (2)	C53—C52—C51	105.6 (10)
O69—Mo16—O42	73.8 (2)	C53—C52—H52A	110.6
O69—Mo16—O52	153.7 (3)	C53—C52—H52B	110.6
O69—Mo16—O64	86.4 (2)	C52—C53—H53A	109.5
O53—Mo17—O42	86.1 (2)	C52—C53—H53B	109.5
O53—Mo17—O65	84.9 (2)	C52—C53—H53C	109.5
O53—Mo17—O70	154.1 (3)	H53A—C53—H53B	109.5
O58—Mo17—O42	170.1 (3)	H53A—C53—H53C	109.5
O58—Mo17—O53	103.5 (3)	H53B—C53—H53C	109.5
O58—Mo17—O64	103.1 (3)	N5—C54—H54A	109.5
O58—Mo17—O65	101.5 (3)	N5—C54—H54B	109.5
O58—Mo17—O70	100.5 (3)	N5—C54—H54C	109.5
O64—Mo17—O42	73.0 (2)	H54A—C54—H54B	109.5
O64—Mo17—O53	97.7 (3)	H54A—C54—H54C	109.5
O64—Mo17—O65	153.9 (2)	H54B—C54—H54C	109.5
O64—Mo17—O70	86.0 (2)	N5—C55—H55A	109.5
O65—Mo17—O42	81.3 (2)	N5—C55—H55B	109.5
O70—Mo17—O42	70.4 (2)	N5—C55—H55C	109.5
O70—Mo17—O65	81.0 (2)	H55A—C55—H55B	109.5
O54—Mo18—O43	81.7 (2)	H55A—C55—H55C	109.5
O54—Mo18—O66	80.8 (2)	H55B—C55—H55C	109.5
O59—Mo18—O43	169.6 (2)	C61—O6S—H6S	109.5
O59—Mo18—O54	101.1 (3)	C61—N6—C64	123.6 (8)
O59—Mo18—O65	103.0 (3)	C61—N6—C65	122.9 (8)
O59—Mo18—O66	99.4 (3)	C65—N6—C64	113.5 (8)
O59—Mo18—O71	102.5 (3)	O6S—C61—C62	120.6 (8)
O65—Mo18—O43	87.1 (2)	N6—C61—O6S	115.7 (9)
O65—Mo18—O54	85.6 (3)	N6—C61—C62	123.7 (9)
O65—Mo18—O66	155.7 (2)	C61—C62—H62A	109.2
O65—Mo18—O71	97.7 (3)	C61—C62—H62B	109.2
O66—Mo18—O43	71.0 (2)	C61—C62—C63	112.2 (8)
O71—Mo18—O43	73.4 (2)	H62A—C62—H62B	107.9
O71—Mo18—O54	154.7 (3)	C63—C62—H62A	109.2
O71—Mo18—O66	86.5 (2)	C63—C62—H62B	109.2
O55—Mo19—O43	87.9 (2)	C62—C63—H63A	109.5
O55—Mo19—O66	98.0 (3)	C62—C63—H63B	109.5
O55—Mo19—O67	85.4 (2)	C62—C63—H63C	109.5
O55—Mo19—O72	156.4 (2)	H63A—C63—H63B	109.5
O60—Mo19—O43	170.0 (3)	H63A—C63—H63C	109.5
O60—Mo19—O55	102.0 (3)	H63B—C63—H63C	109.5
O60—Mo19—O66	102.6 (3)	N6—C64—H64A	109.5
O60—Mo19—O67	100.2 (3)	N6—C64—H64B	109.5
O60—Mo19—O72	99.2 (3)	N6—C64—H64C	109.5
O66—Mo19—O43	74.5 (2)	H64A—C64—H64B	109.5
O66—Mo19—O67	155.7 (3)	H64A—C64—H64C	109.5
O66—Mo19—O72	87.1 (2)	H64B—C64—H64C	109.5
O67—Mo19—O43	81.6 (2)	N6—C65—H65A	109.5
O72—Mo19—O43	71.2 (2)	N6—C65—H65B	109.5
O72—Mo19—O67	80.9 (2)	N6—C65—H65C	109.5
O56—Mo20—O44	81.5 (2)	H65A—C65—H65B	109.5
O56—Mo20—O68	80.0 (2)	H65A—C65—H65C	109.5
O61—Mo20—O44	169.8 (3)	H65B—C65—H65C	109.5
O61—Mo20—O56	102.0 (3)	C91—O9S—H9S	120
O61—Mo20—O67	103.4 (3)	C91—N9—C94	119.5 (11)
O61—Mo20—O68	100.9 (3)	C91—N9—C95	124.6 (12)
O61—Mo20—O73	102.3 (3)	C94—N9—C95	115.9 (11)
O67—Mo20—O44	86.4 (2)	O9S—C91—N9	119.1 (11)
O67—Mo20—O56	84.8 (2)	O9S—C91—C92	118.6 (11)
O67—Mo20—O68	153.5 (3)	N9—C91—C92	122.3 (11)
O67—Mo20—O73	98.3 (3)	C91—C92—H92A	108.8
O68—Mo20—O44	70.0 (2)	C91—C92—H92B	108.8
O73—Mo20—O44	73.1 (2)	H92A—C92—H92B	107.7
O73—Mo20—O56	154.1 (3)	C93—C92—C91	113.6 (9)
O73—Mo20—O68	86.6 (3)	C93—C92—H92A	108.8
O51—Mo21—O44	87.2 (2)	C93—C92—H92B	108.8
O51—Mo21—O63	85.0 (2)	C92—C93—H93A	109.5
O51—Mo21—O68	97.5 (3)	C92—C93—H93B	109.5
O51—Mo21—O74	155.5 (3)	C92—C93—H93C	109.5
O62—Mo21—O44	169.7 (3)	H93A—C93—H93B	109.5
O62—Mo21—O51	102.4 (3)	H93A—C93—H93C	109.5
O62—Mo21—O63	102.3 (3)	H93B—C93—H93C	109.5
O62—Mo21—O68	102.3 (3)	N9—C94—H94A	109.5
O62—Mo21—O74	100.3 (3)	N9—C94—H94B	109.5
O63—Mo21—O44	82.0 (2)	N9—C94—H94C	109.5
O63—Mo21—O74	81.1 (2)	H94A—C94—H94B	109.5
O68—Mo21—O44	72.4 (2)	H94A—C94—H94C	109.5
O68—Mo21—O63	154.1 (3)	H94B—C94—H94C	109.5
O68—Mo21—O74	86.6 (3)	N9—C95—H95A	109.5
O74—Mo21—O44	71.0 (2)	N9—C95—H95B	109.5
O69—Mo22—O42	71.3 (2)	N9—C95—H95C	109.5
O70—Mo22—O42	72.9 (2)	H95A—C95—H95B	109.5
O70—Mo22—O69	86.6 (2)	H95A—C95—H95C	109.5
O70—Mo22—O75	154.1 (3)	H95B—C95—H95C	109.5
O75—Mo22—O42	81.6 (2)	C101—O10S—H10S	116
O75—Mo22—O69	80.9 (2)	C101—N10—C104	119.6 (9)
O76—Mo22—O42	86.1 (2)	C101—N10—C105	123.0 (9)
O76—Mo22—O69	154.8 (3)	C105—N10—C104	117.0 (9)
O76—Mo22—O70	97.6 (3)	O10S—C101—N10	117.4 (10)
O76—Mo22—O75	84.9 (2)	O10S—C101—C102	120.7 (9)
O78—Mo22—O42	169.0 (3)	N10—C101—C102	121.9 (10)
O78—Mo22—O69	98.5 (3)	C101—C102—H10A	109.3
O78—Mo22—O70	102.9 (3)	C101—C102—H10B	109.3
O78—Mo22—O75	101.4 (3)	C101—C102—C103	111.7 (9)
O78—Mo22—O76	104.6 (3)	H10A—C102—H10B	107.9
O71—Mo23—O43	71.3 (2)	C103—C102—H10A	109.3
O72—Mo23—O43	73.2 (2)	C103—C102—H10B	109.3
O72—Mo23—O71	86.5 (2)	C102—C103—H10C	109.5
O72—Mo23—O76	154.7 (3)	C102—C103—H10D	109.5
O76—Mo23—O43	81.9 (2)	C102—C103—H10E	109.5
O76—Mo23—O71	81.2 (2)	H10C—C103—H10D	109.5
O77—Mo23—O43	87.3 (2)	H10C—C103—H10E	109.5
O77—Mo23—O71	156.1 (2)	H10D—C103—H10E	109.5
O77—Mo23—O72	97.6 (3)	N10—C104—H10F	109.5
O77—Mo23—O76	85.5 (2)	N10—C104—H10G	109.5
O79—Mo23—O43	168.9 (2)	N10—C104—H10H	109.5
O79—Mo23—O71	98.1 (3)	H10F—C104—H10G	109.5
O79—Mo23—O72	103.5 (3)	H10F—C104—H10H	109.5
O79—Mo23—O76	100.1 (3)	H10G—C104—H10H	109.5
O79—Mo23—O77	103.8 (3)	N10—C105—H10I	109.5
O73—Mo24—O44	70.0 (2)	N10—C105—H10J	109.5
O73—Mo24—O77	80.4 (2)	N10—C105—H10K	109.5
O74—Mo24—O44	73.5 (2)	H10I—C105—H10J	109.5
O74—Mo24—O73	86.6 (3)	H10I—C105—H10K	109.5
O74—Mo24—O77	154.2 (3)	H10J—C105—H10K	109.5
O75—Mo24—O44	87.2 (2)		
			
O1—Mo1—O8—Mo3	−2.5 (3)	O43—Mo18—O66—Mo19	−1.9 (3)
O1—Mo1—O9—Mo2	0.1 (3)	O43—Mo18—O71—Mo23	1.4 (3)
O1—Mo1—O11—Mo9	53.7 (6)	O43—Mo19—O55—Mo15	−48.9 (6)
O1—Mo1—O12—Mo4	−57.3 (6)	O43—Mo19—O66—Mo18	1.9 (3)
O1—Mo2—O9—Mo1	−0.1 (3)	O43—Mo19—O67—Mo20	55.1 (7)
O1—Mo2—O10—Mo3	0.4 (3)	O43—Mo19—O72—Mo23	−2.2 (3)
O1—Mo2—O13—Mo5	53.0 (6)	O43—Mo23—O71—Mo18	−1.4 (3)
O1—Mo2—O14—Mo6	−58.5 (7)	O43—Mo23—O72—Mo19	2.2 (3)
O1—Mo3—O8—Mo1	2.5 (3)	O43—Mo23—O76—Mo22	55.2 (7)
O1—Mo3—O10—Mo2	−0.4 (3)	O43—Mo23—O77—Mo24	−50.6 (7)
O1—Mo3—O15—Mo7	50.6 (6)	O43—P2—O41—Mo13	−173.0 (3)
O1—Mo3—O16—Mo8	−58.5 (7)	O43—P2—O41—Mo14	−55.7 (5)
O1—P1—O2—Mo4	54.3 (4)	O43—P2—O41—Mo15	65.9 (4)
O1—P1—O2—Mo5	−65.3 (4)	O43—P2—O42—Mo16	−174.3 (3)
O1—P1—O2—Mo10	173.0 (3)	O43—P2—O42—Mo17	66.0 (5)
O1—P1—O3—Mo6	54.1 (4)	O43—P2—O42—Mo22	−54.8 (5)
O1—P1—O3—Mo7	−66.8 (4)	O43—P2—O44—Mo20	−53.0 (5)
O1—P1—O3—Mo11	173.7 (3)	O43—P2—O44—Mo21	−174.3 (3)
O1—P1—O4—Mo8	53.6 (4)	O43—P2—O44—Mo24	67.6 (4)
O1—P1—O4—Mo9	−66.5 (4)	O44—Mo20—O56—Mo15	54.4 (7)
O1—P1—O4—Mo12	173.9 (3)	O44—Mo20—O67—Mo19	−49.7 (7)
O2—Mo4—O12—Mo1	52.7 (6)	O44—Mo20—O68—Mo21	−3.0 (3)
O2—Mo4—O23—Mo9	−56.2 (6)	O44—Mo20—O73—Mo24	2.2 (3)
O2—Mo4—O24—Mo5	−2.5 (3)	O44—Mo21—O51—Mo13	−49.7 (7)
O2—Mo4—O29—Mo10	2.4 (3)	O44—Mo21—O63—Mo16	55.1 (7)
O2—Mo5—O13—Mo2	−57.8 (6)	O44—Mo21—O68—Mo20	2.9 (3)
O2—Mo5—O24—Mo4	2.6 (3)	O44—Mo21—O74—Mo24	−1.6 (3)
O2—Mo5—O25—Mo6	50.1 (7)	O44—Mo24—O73—Mo20	−2.3 (3)
O2—Mo5—O30—Mo10	−2.6 (3)	O44—Mo24—O74—Mo21	1.5 (3)
O2—Mo10—O29—Mo4	−2.4 (3)	O44—Mo24—O75—Mo22	−49.2 (7)
O2—Mo10—O30—Mo5	2.6 (3)	O44—Mo24—O77—Mo23	55.7 (7)
O2—Mo10—O35—Mo12	51.5 (6)	O44—P2—O41—Mo13	68.4 (4)
O2—Mo10—O36—Mo11	−56.7 (7)	O44—P2—O41—Mo14	−174.3 (3)
O2—P1—O1—Mo1	−65.6 (4)	O44—P2—O41—Mo15	−52.6 (5)
O2—P1—O1—Mo2	52.7 (4)	O44—P2—O42—Mo16	−54.1 (5)
O2—P1—O1—Mo3	172.1 (3)	O44—P2—O42—Mo17	−173.8 (3)
O2—P1—O3—Mo6	−66.1 (4)	O44—P2—O42—Mo22	65.4 (5)
O2—P1—O3—Mo7	173.0 (3)	O44—P2—O43—Mo18	−174.5 (3)
O2—P1—O3—Mo11	53.6 (4)	O44—P2—O43—Mo19	65.2 (5)
O2—P1—O4—Mo8	174.8 (3)	O44—P2—O43—Mo23	−55.3 (5)
O2—P1—O4—Mo9	54.7 (4)	O45—Mo13—O41—Mo14	68.8 (13)
O2—P1—O4—Mo12	−65.0 (4)	O45—Mo13—O41—Mo15	−21.4 (14)
O3—Mo6—O14—Mo2	53.0 (7)	O45—Mo13—O41—P2	−157.8 (11)
O3—Mo6—O25—Mo5	−55.1 (7)	O45—Mo13—O48—Mo15	173.7 (4)
O3—Mo6—O26—Mo7	−1.9 (3)	O45—Mo13—O49—Mo14	−166.8 (4)
O3—Mo6—O31—Mo11	2.9 (3)	O45—Mo13—O51—Mo21	−136.0 (7)
O3—Mo7—O15—Mo3	−56.1 (6)	O45—Mo13—O52—Mo16	128.7 (7)
O3—Mo7—O26—Mo6	1.9 (3)	O46—Mo14—O41—Mo13	−25.6 (15)
O3—Mo7—O27—Mo8	49.8 (7)	O46—Mo14—O41—Mo15	64.5 (15)
O3—Mo7—O32—Mo11	−2.8 (3)	O46—Mo14—O41—P2	−158.7 (13)
O3—Mo11—O31—Mo6	−2.8 (3)	O46—Mo14—O49—Mo13	172.5 (4)
O3—Mo11—O32—Mo7	2.9 (3)	O46—Mo14—O50—Mo15	−168.2 (4)
O3—Mo11—O36—Mo10	51.2 (7)	O46—Mo14—O53—Mo17	−134.5 (7)
O3—Mo11—O37—Mo12	−56.2 (7)	O46—Mo14—O54—Mo18	132.6 (7)
O3—P1—O1—Mo1	175.4 (3)	O47—Mo15—O41—Mo13	65.5 (13)
O3—P1—O1—Mo2	−66.3 (4)	O47—Mo15—O41—Mo14	−23.4 (13)
O3—P1—O1—Mo3	53.1 (4)	O47—Mo15—O41—P2	−159.3 (11)
O3—P1—O2—Mo4	173.8 (3)	O47—Mo15—O48—Mo13	−167.3 (4)
O3—P1—O2—Mo5	54.3 (4)	O47—Mo15—O50—Mo14	173.4 (4)
O3—P1—O2—Mo10	−67.5 (4)	O47—Mo15—O55—Mo19	−137.1 (7)
O3—P1—O4—Mo8	−66.1 (4)	O47—Mo15—O56—Mo20	133.4 (7)
O3—P1—O4—Mo9	173.8 (3)	O48—Mo13—O41—Mo14	91.4 (2)
O3—P1—O4—Mo12	54.1 (4)	O48—Mo13—O41—Mo15	1.2 (2)
O4—Mo8—O16—Mo3	52.7 (7)	O48—Mo13—O41—P2	−135.2 (4)
O4—Mo8—O27—Mo7	−55.1 (7)	O48—Mo13—O49—Mo14	−67.8 (4)
O4—Mo8—O28—Mo9	−3.9 (3)	O48—Mo13—O51—Mo21	126.2 (7)
O4—Mo8—O33—Mo12	1.0 (3)	O48—Mo13—O52—Mo16	−27.5 (11)
O4—Mo9—O11—Mo1	−58.0 (6)	O48—Mo15—O41—Mo13	−1.3 (2)
O4—Mo9—O23—Mo4	50.9 (6)	O48—Mo15—O41—Mo14	−90.1 (2)
O4—Mo9—O28—Mo8	3.9 (3)	O48—Mo15—O41—P2	134.0 (4)
O4—Mo9—O34—Mo12	−2.1 (3)	O48—Mo15—O50—Mo14	71.9 (4)
O4—Mo12—O33—Mo8	−1.0 (3)	O48—Mo15—O55—Mo19	67.7 (9)
O4—Mo12—O34—Mo9	2.1 (3)	O48—Mo15—O56—Mo20	−122.1 (7)
O4—Mo12—O35—Mo10	−57.2 (6)	O49—Mo13—O41—Mo14	−1.7 (2)
O4—Mo12—O37—Mo11	51.2 (7)	O49—Mo13—O41—Mo15	−91.8 (2)
O4—P1—O1—Mo1	55.4 (4)	O49—Mo13—O41—P2	131.7 (4)
O4—P1—O1—Mo2	173.7 (3)	O49—Mo13—O48—Mo15	72.0 (4)
O4—P1—O1—Mo3	−66.9 (4)	O49—Mo13—O51—Mo21	64.2 (10)
O4—P1—O2—Mo4	−66.3 (4)	O49—Mo13—O52—Mo16	−126.9 (7)
O4—P1—O2—Mo5	174.2 (3)	O49—Mo14—O41—Mo13	1.5 (2)
O4—P1—O2—Mo10	52.4 (4)	O49—Mo14—O41—Mo15	91.6 (2)
O4—P1—O3—Mo6	173.9 (3)	O49—Mo14—O41—P2	−131.6 (5)
O4—P1—O3—Mo7	53.0 (4)	O49—Mo14—O50—Mo15	−69.2 (4)
O4—P1—O3—Mo11	−66.5 (4)	O49—Mo14—O53—Mo17	127.9 (7)
O5—Mo1—O8—Mo3	168.3 (3)	O49—Mo14—O54—Mo18	−21.9 (12)
O5—Mo1—O9—Mo2	−175.6 (3)	O50—Mo14—O41—Mo13	−91.3 (2)
O5—Mo1—O11—Mo9	−129.0 (6)	O50—Mo14—O41—Mo15	−1.2 (2)
O5—Mo1—O12—Mo4	132.3 (6)	O50—Mo14—O41—P2	135.6 (5)
O6—Mo2—O9—Mo1	170.9 (3)	O50—Mo14—O49—Mo13	70.3 (4)
O6—Mo2—O10—Mo3	−176.8 (3)	O50—Mo14—O53—Mo17	66.1 (10)
O6—Mo2—O13—Mo5	−128.2 (6)	O50—Mo14—O54—Mo18	−122.3 (7)
O6—Mo2—O14—Mo6	130.7 (7)	O50—Mo15—O41—Mo13	90.0 (2)
O7—Mo3—O1—Mo1	10.5 (14)	O50—Mo15—O41—Mo14	1.1 (2)
O7—Mo3—O1—Mo2	−78.3 (13)	O50—Mo15—O41—P2	−134.8 (4)
O7—Mo3—O1—P1	147.1 (12)	O50—Mo15—O48—Mo13	−69.5 (4)
O7—Mo3—O8—Mo1	−175.3 (3)	O50—Mo15—O55—Mo19	126.0 (7)
O7—Mo3—O10—Mo2	169.2 (3)	O50—Mo15—O56—Mo20	−23.4 (12)
O7—Mo3—O15—Mo7	−129.6 (6)	O51—Mo13—O41—Mo14	174.3 (2)
O7—Mo3—O16—Mo8	132.0 (7)	O51—Mo13—O41—Mo15	84.1 (2)
O8—Mo1—O1—Mo2	91.1 (2)	O51—Mo13—O41—P2	−52.3 (4)
O8—Mo1—O1—Mo3	1.77 (18)	O51—Mo13—O48—Mo15	−86.3 (4)
O8—Mo1—O1—P1	−134.8 (4)	O51—Mo13—O49—Mo14	−7.1 (8)
O8—Mo1—O9—Mo2	−74.3 (3)	O51—Mo13—O52—Mo16	28.4 (7)
O8—Mo1—O11—Mo9	127.0 (6)	O51—Mo21—O44—Mo20	−100.7 (2)
O8—Mo1—O12—Mo4	−69.1 (9)	O51—Mo21—O44—Mo24	169.6 (2)
O8—Mo3—O1—Mo1	−1.66 (17)	O51—Mo21—O44—P2	35.9 (4)
O8—Mo3—O1—Mo2	−90.4 (2)	O51—Mo21—O63—Mo16	−32.8 (7)
O8—Mo3—O1—P1	134.9 (4)	O51—Mo21—O68—Mo20	87.5 (4)
O8—Mo3—O10—Mo2	72.1 (3)	O51—Mo21—O74—Mo24	−29.9 (9)
O8—Mo3—O15—Mo7	23.8 (11)	O52—Mo13—O41—Mo14	−100.3 (2)
O8—Mo3—O16—Mo8	−131.8 (7)	O52—Mo13—O41—Mo15	169.5 (2)
O9—Mo1—O1—Mo2	−0.06 (18)	O52—Mo13—O41—P2	33.1 (4)
O9—Mo1—O1—Mo3	−89.4 (2)	O52—Mo13—O48—Mo15	−29.6 (8)
O9—Mo1—O1—P1	134.0 (4)	O52—Mo13—O49—Mo14	86.7 (4)
O9—Mo1—O8—Mo3	70.1 (3)	O52—Mo13—O51—Mo21	−32.4 (7)
O9—Mo1—O11—Mo9	30.7 (11)	O52—Mo16—O42—Mo17	84.3 (2)
O9—Mo1—O12—Mo4	−130.4 (7)	O52—Mo16—O42—Mo22	173.4 (2)
O9—Mo2—O1—Mo1	0.06 (19)	O52—Mo16—O42—P2	−51.2 (4)
O9—Mo2—O1—Mo3	91.0 (2)	O52—Mo16—O63—Mo21	30.5 (7)
O9—Mo2—O1—P1	−134.2 (4)	O52—Mo16—O64—Mo17	−85.3 (4)
O9—Mo2—O10—Mo3	−74.4 (3)	O52—Mo16—O69—Mo22	−8.7 (8)
O9—Mo2—O13—Mo5	126.7 (6)	O53—Mo14—O41—Mo13	84.0 (2)
O9—Mo2—O14—Mo6	−71.2 (9)	O53—Mo14—O41—Mo15	174.1 (2)
O10—Mo2—O1—Mo1	−91.2 (2)	O53—Mo14—O41—P2	−49.1 (4)
O10—Mo2—O1—Mo3	−0.25 (17)	O53—Mo14—O49—Mo13	−86.9 (4)
O10—Mo2—O1—P1	134.6 (4)	O53—Mo14—O50—Mo15	−8.8 (8)
O10—Mo2—O9—Mo1	71.6 (3)	O53—Mo14—O54—Mo18	31.6 (8)
O10—Mo2—O13—Mo5	28.3 (10)	O53—Mo17—O42—Mo16	−100.6 (2)
O10—Mo2—O14—Mo6	−130.9 (7)	O53—Mo17—O42—Mo22	170.4 (2)
O10—Mo3—O1—Mo1	89.0 (2)	O53—Mo17—O42—P2	34.2 (4)
O10—Mo3—O1—Mo2	0.27 (19)	O53—Mo17—O64—Mo16	85.6 (4)
O10—Mo3—O1—P1	−134.4 (4)	O53—Mo17—O65—Mo18	−31.5 (7)
O10—Mo3—O8—Mo1	−72.6 (3)	O53—Mo17—O70—Mo22	−28.5 (8)
O10—Mo3—O15—Mo7	124.6 (6)	O54—Mo14—O41—Mo13	169.1 (2)
O10—Mo3—O16—Mo8	−72.0 (9)	O54—Mo14—O41—Mo15	−100.8 (2)
O11—Mo1—O1—Mo2	−170.5 (2)	O54—Mo14—O41—P2	36.0 (4)
O11—Mo1—O1—Mo3	100.2 (2)	O54—Mo14—O49—Mo13	−32.6 (8)
O11—Mo1—O1—P1	−36.4 (4)	O54—Mo14—O50—Mo15	85.9 (4)
O11—Mo1—O8—Mo3	−86.1 (3)	O54—Mo14—O53—Mo17	−31.7 (7)
O11—Mo1—O9—Mo2	24.3 (8)	O54—Mo18—O43—Mo19	84.3 (2)
O11—Mo1—O12—Mo4	29.0 (6)	O54—Mo18—O43—Mo23	174.2 (2)
O11—Mo9—O4—Mo8	−85.04 (19)	O54—Mo18—O43—P2	−51.3 (4)
O11—Mo9—O4—Mo12	−174.4 (2)	O54—Mo18—O65—Mo17	31.3 (7)
O11—Mo9—O4—P1	50.6 (4)	O54—Mo18—O66—Mo19	−86.2 (4)
O11—Mo9—O23—Mo4	−29.7 (6)	O54—Mo18—O71—Mo23	−9.9 (8)
O11—Mo9—O28—Mo8	87.0 (4)	O55—Mo15—O41—Mo13	172.6 (2)
O11—Mo9—O34—Mo12	7.4 (8)	O55—Mo15—O41—Mo14	83.8 (2)
O12—Mo1—O1—Mo2	−83.7 (2)	O55—Mo15—O41—P2	−52.1 (4)
O12—Mo1—O1—Mo3	−173.1 (2)	O55—Mo15—O48—Mo13	−12.3 (8)
O12—Mo1—O1—P1	50.3 (4)	O55—Mo15—O50—Mo14	−86.8 (4)
O12—Mo1—O8—Mo3	9.7 (7)	O55—Mo15—O56—Mo20	32.9 (7)
O12—Mo1—O9—Mo2	84.1 (3)	O55—Mo19—O43—Mo18	−100.2 (2)
O12—Mo1—O11—Mo9	−28.3 (6)	O55—Mo19—O43—Mo23	170.2 (2)
O12—Mo4—O2—Mo5	100.3 (2)	O55—Mo19—O43—P2	35.0 (4)
O12—Mo4—O2—Mo10	−170.5 (2)	O55—Mo19—O66—Mo18	87.5 (4)
O12—Mo4—O2—P1	−35.4 (4)	O55—Mo19—O67—Mo20	−33.5 (7)
O12—Mo4—O23—Mo9	30.7 (6)	O55—Mo19—O72—Mo23	−31.1 (8)
O12—Mo4—O24—Mo5	−86.4 (4)	O56—Mo15—O41—Mo13	−100.8 (2)
O12—Mo4—O29—Mo10	29.3 (8)	O56—Mo15—O41—Mo14	170.4 (2)
O13—Mo2—O1—Mo1	99.5 (2)	O56—Mo15—O41—P2	34.5 (4)
O13—Mo2—O1—Mo3	−169.6 (2)	O56—Mo15—O48—Mo13	87.0 (4)
O13—Mo2—O1—P1	−34.7 (4)	O56—Mo15—O50—Mo14	−29.5 (9)
O13—Mo2—O9—Mo1	−83.8 (3)	O56—Mo15—O55—Mo19	−34.2 (7)
O13—Mo2—O10—Mo3	26.5 (7)	O56—Mo20—O44—Mo21	84.2 (2)
O13—Mo2—O14—Mo6	28.4 (7)	O56—Mo20—O44—Mo24	173.5 (2)
O13—Mo5—O2—Mo4	−83.9 (2)	O56—Mo20—O44—P2	−51.4 (4)
O13—Mo5—O2—Mo10	−172.8 (2)	O56—Mo20—O67—Mo19	32.1 (7)
O13—Mo5—O2—P1	50.5 (4)	O56—Mo20—O68—Mo21	−87.5 (4)
O13—Mo5—O24—Mo4	86.0 (3)	O56—Mo20—O73—Mo24	−9.2 (8)
O13—Mo5—O25—Mo6	−30.9 (7)	O57—Mo16—O42—Mo17	−18.7 (14)
O13—Mo5—O30—Mo10	10.3 (8)	O57—Mo16—O42—Mo22	70.4 (13)
O14—Mo2—O1—Mo1	−174.3 (2)	O57—Mo16—O42—P2	−154.2 (12)
O14—Mo2—O1—Mo3	−83.4 (2)	O57—Mo16—O52—Mo13	−132.6 (7)
O14—Mo2—O1—P1	51.4 (4)	O57—Mo16—O63—Mo21	131.8 (7)
O14—Mo2—O9—Mo1	12.9 (7)	O57—Mo16—O64—Mo17	174.0 (4)
O14—Mo2—O10—Mo3	83.8 (3)	O57—Mo16—O69—Mo22	−166.6 (4)
O14—Mo2—O13—Mo5	−28.1 (6)	O58—Mo17—O53—Mo14	131.4 (7)
O14—Mo6—O3—Mo7	101.2 (2)	O58—Mo17—O64—Mo16	−168.5 (4)
O14—Mo6—O3—Mo11	−170.0 (2)	O58—Mo17—O65—Mo18	−134.3 (7)
O14—Mo6—O3—P1	−34.8 (4)	O58—Mo17—O70—Mo22	173.6 (4)
O14—Mo6—O25—Mo5	31.3 (7)	O59—Mo18—O43—Mo19	−22.3 (15)
O14—Mo6—O26—Mo7	−84.8 (4)	O59—Mo18—O43—Mo23	67.6 (14)
O14—Mo6—O31—Mo11	30.6 (8)	O59—Mo18—O43—P2	−157.9 (13)
O15—Mo3—O1—Mo1	−170.9 (2)	O59—Mo18—O54—Mo14	−135.2 (7)
O15—Mo3—O1—Mo2	100.3 (2)	O59—Mo18—O65—Mo17	131.7 (7)
O15—Mo3—O1—P1	−34.4 (4)	O59—Mo18—O66—Mo19	173.9 (4)
O15—Mo3—O8—Mo1	30.8 (8)	O59—Mo18—O71—Mo23	−168.7 (4)
O15—Mo3—O10—Mo2	−85.2 (3)	O60—Mo19—O55—Mo15	132.3 (6)
O15—Mo3—O16—Mo8	29.6 (7)	O60—Mo19—O66—Mo18	−168.2 (4)
O15—Mo7—O3—Mo6	−83.8 (2)	O60—Mo19—O67—Mo20	−134.9 (7)
O15—Mo7—O3—Mo11	−172.7 (2)	O60—Mo19—O72—Mo23	174.7 (4)
O15—Mo7—O3—P1	52.3 (4)	O61—Mo20—O44—Mo21	−26.4 (15)
O15—Mo7—O26—Mo6	85.6 (4)	O61—Mo20—O44—Mo24	62.9 (14)
O15—Mo7—O27—Mo8	−31.4 (7)	O61—Mo20—O44—P2	−162.0 (12)
O15—Mo7—O32—Mo11	9.9 (8)	O61—Mo20—O56—Mo15	−135.4 (7)
O16—Mo3—O1—Mo1	−84.9 (2)	O61—Mo20—O67—Mo19	133.2 (7)
O16—Mo3—O1—Mo2	−173.7 (2)	O61—Mo20—O68—Mo21	172.1 (4)
O16—Mo3—O1—P1	51.6 (4)	O61—Mo20—O73—Mo24	−168.3 (4)
O16—Mo3—O8—Mo1	85.1 (3)	O62—Mo21—O44—Mo20	58.1 (14)
O16—Mo3—O10—Mo2	13.3 (7)	O62—Mo21—O44—Mo24	−31.5 (14)
O16—Mo3—O15—Mo7	−29.7 (6)	O62—Mo21—O44—P2	−165.3 (12)
O16—Mo8—O4—Mo9	101.8 (2)	O62—Mo21—O51—Mo13	134.1 (7)
O16—Mo8—O4—Mo12	−168.9 (2)	O62—Mo21—O63—Mo16	−134.4 (7)
O16—Mo8—O4—P1	−33.7 (4)	O62—Mo21—O68—Mo20	−168.0 (4)
O16—Mo8—O27—Mo7	31.0 (7)	O62—Mo21—O74—Mo24	172.9 (4)
O16—Mo8—O28—Mo9	−86.7 (4)	O63—Mo16—O42—Mo17	170.3 (2)
O16—Mo8—O33—Mo12	28.5 (8)	O63—Mo16—O42—Mo22	−100.6 (2)
O17—Mo4—O12—Mo1	−129.4 (6)	O63—Mo16—O42—P2	34.8 (4)
O17—Mo4—O23—Mo9	133.1 (6)	O63—Mo16—O52—Mo13	−28.8 (8)
O17—Mo4—O24—Mo5	168.4 (3)	O63—Mo16—O64—Mo17	−28.4 (8)
O17—Mo4—O29—Mo10	−173.7 (4)	O63—Mo16—O69—Mo22	86.7 (4)
O18—Mo5—O13—Mo2	131.6 (6)	O63—Mo21—O44—Mo20	173.9 (2)
O18—Mo5—O24—Mo4	−174.5 (4)	O63—Mo21—O44—Mo24	84.2 (2)
O18—Mo5—O25—Mo6	−130.8 (7)	O63—Mo21—O44—P2	−49.5 (4)
O18—Mo5—O30—Mo10	168.3 (4)	O63—Mo21—O51—Mo13	32.6 (7)
O19—Mo6—O14—Mo2	−127.7 (7)	O63—Mo21—O68—Mo20	−6.6 (9)
O19—Mo6—O25—Mo5	133.8 (7)	O63—Mo21—O74—Mo24	−86.2 (4)
O19—Mo6—O26—Mo7	169.4 (4)	O64—Mo16—O42—Mo17	1.2 (2)
O19—Mo6—O31—Mo11	−174.6 (4)	O64—Mo16—O42—Mo22	90.4 (2)
O20—Mo7—O3—Mo6	11.5 (14)	O64—Mo16—O42—P2	−134.2 (4)
O20—Mo7—O3—Mo11	−77.4 (13)	O64—Mo16—O52—Mo13	130.1 (8)
O20—Mo7—O3—P1	147.6 (12)	O64—Mo16—O63—Mo21	−25.3 (12)
O20—Mo7—O15—Mo3	134.6 (7)	O64—Mo16—O69—Mo22	−68.1 (4)
O20—Mo7—O26—Mo6	−175.8 (4)	O64—Mo17—O42—Mo16	−1.3 (2)
O20—Mo7—O27—Mo8	−130.4 (7)	O64—Mo17—O42—Mo22	−90.4 (2)
O20—Mo7—O32—Mo11	166.4 (4)	O64—Mo17—O42—P2	133.5 (4)
O21—Mo8—O16—Mo3	−128.7 (7)	O64—Mo17—O53—Mo14	−123.1 (7)
O21—Mo8—O27—Mo7	133.4 (7)	O64—Mo17—O65—Mo18	65.5 (10)
O21—Mo8—O28—Mo9	167.7 (3)	O64—Mo17—O70—Mo22	70.9 (4)
O21—Mo8—O33—Mo12	−175.9 (4)	O65—Mo17—O42—Mo16	174.0 (2)
O22—Mo9—O11—Mo1	131.2 (7)	O65—Mo17—O42—Mo22	85.0 (2)
O22—Mo9—O23—Mo4	−129.7 (6)	O65—Mo17—O42—P2	−51.2 (4)
O22—Mo9—O28—Mo8	−173.5 (4)	O65—Mo17—O53—Mo14	30.8 (7)
O22—Mo9—O34—Mo12	168.7 (4)	O65—Mo17—O64—Mo16	−8.5 (8)
O23—Mo4—O2—Mo5	−174.0 (2)	O65—Mo17—O70—Mo22	−86.3 (4)
O23—Mo4—O2—Mo10	−84.8 (2)	O65—Mo18—O43—Mo19	170.3 (2)
O23—Mo4—O2—P1	50.3 (4)	O65—Mo18—O43—Mo23	−99.8 (2)
O23—Mo4—O12—Mo1	−28.5 (6)	O65—Mo18—O43—P2	34.7 (4)
O23—Mo4—O24—Mo5	7.4 (7)	O65—Mo18—O54—Mo14	−32.9 (8)
O23—Mo4—O29—Mo10	85.9 (4)	O65—Mo18—O66—Mo19	−29.2 (8)
O23—Mo9—O4—Mo8	−169.9 (2)	O65—Mo18—O71—Mo23	86.1 (4)
O23—Mo9—O4—Mo12	100.7 (2)	O66—Mo18—O43—Mo19	1.2 (2)
O23—Mo9—O4—P1	−34.2 (4)	O66—Mo18—O43—Mo23	91.1 (2)
O23—Mo9—O11—Mo1	28.7 (6)	O66—Mo18—O43—P2	−134.4 (4)
O23—Mo9—O28—Mo8	33.7 (7)	O66—Mo18—O54—Mo14	126.9 (8)
O23—Mo9—O34—Mo12	−85.5 (4)	O66—Mo18—O65—Mo17	−24.9 (12)
O24—Mo4—O2—Mo5	1.68 (19)	O66—Mo18—O71—Mo23	−69.8 (4)
O24—Mo4—O2—Mo10	90.9 (2)	O66—Mo19—O43—Mo18	−1.3 (2)
O24—Mo4—O2—P1	−134.1 (4)	O66—Mo19—O43—Mo23	−90.9 (2)
O24—Mo4—O12—Mo1	125.5 (6)	O66—Mo19—O43—P2	133.9 (4)
O24—Mo4—O23—Mo9	−65.8 (9)	O66—Mo19—O55—Mo15	−123.0 (7)
O24—Mo4—O29—Mo10	−71.2 (4)	O66—Mo19—O67—Mo20	65.9 (10)
O24—Mo5—O2—Mo4	−1.54 (18)	O66—Mo19—O72—Mo23	72.4 (4)
O24—Mo5—O2—Mo10	−90.4 (2)	O67—Mo19—O43—Mo18	174.1 (2)
O24—Mo5—O2—P1	132.9 (4)	O67—Mo19—O43—Mo23	84.6 (2)
O24—Mo5—O13—Mo2	−130.6 (6)	O67—Mo19—O43—P2	−50.6 (4)
O24—Mo5—O25—Mo6	21.5 (11)	O67—Mo19—O55—Mo15	32.8 (7)
O24—Mo5—O30—Mo10	69.2 (4)	O67—Mo19—O66—Mo18	−9.2 (8)
O25—Mo5—O2—Mo4	−169.1 (2)	O67—Mo19—O72—Mo23	−86.4 (4)
O25—Mo5—O2—Mo10	102.1 (2)	O67—Mo20—O44—Mo21	169.5 (2)
O25—Mo5—O2—P1	−34.7 (4)	O67—Mo20—O44—Mo24	−101.2 (2)
O25—Mo5—O13—Mo2	29.3 (6)	O67—Mo20—O44—P2	33.9 (4)
O25—Mo5—O24—Mo4	32.8 (7)	O67—Mo20—O56—Mo15	−32.7 (7)
O25—Mo5—O30—Mo10	−86.2 (4)	O67—Mo20—O68—Mo21	−31.4 (8)
O25—Mo6—O3—Mo7	−173.5 (2)	O67—Mo20—O73—Mo24	85.9 (4)
O25—Mo6—O3—Mo11	−84.6 (2)	O68—Mo20—O44—Mo21	1.8 (2)
O25—Mo6—O3—P1	50.5 (4)	O68—Mo20—O44—Mo24	91.1 (2)
O25—Mo6—O14—Mo2	−27.7 (7)	O68—Mo20—O44—P2	−133.8 (5)
O25—Mo6—O26—Mo7	9.9 (8)	O68—Mo20—O56—Mo15	125.5 (7)
O25—Mo6—O31—Mo11	86.0 (4)	O68—Mo20—O67—Mo19	−23.0 (11)
O26—Mo6—O3—Mo7	1.3 (2)	O68—Mo20—O73—Mo24	−67.9 (4)
O26—Mo6—O3—Mo11	90.2 (2)	O68—Mo21—O44—Mo20	−1.9 (2)
O26—Mo6—O3—P1	−134.7 (4)	O68—Mo21—O44—Mo24	−91.6 (2)
O26—Mo6—O14—Mo2	126.1 (7)	O68—Mo21—O44—P2	134.7 (4)
O26—Mo6—O25—Mo5	−66.6 (10)	O68—Mo21—O51—Mo13	−121.5 (7)
O26—Mo6—O31—Mo11	−71.4 (4)	O68—Mo21—O63—Mo16	64.2 (10)
O26—Mo7—O3—Mo6	−1.15 (18)	O68—Mo21—O74—Mo24	70.9 (4)
O26—Mo7—O3—Mo11	−90.0 (2)	O69—Mo16—O42—Mo17	−90.8 (2)
O26—Mo7—O3—P1	134.9 (4)	O69—Mo16—O42—Mo22	−1.6 (2)
O26—Mo7—O15—Mo3	−128.5 (7)	O69—Mo16—O42—P2	133.8 (4)
O26—Mo7—O27—Mo8	22.6 (11)	O69—Mo16—O52—Mo13	69.4 (10)
O26—Mo7—O32—Mo11	68.6 (4)	O69—Mo16—O63—Mo21	−123.2 (7)
O27—Mo7—O3—Mo6	−169.4 (2)	O69—Mo16—O64—Mo17	72.0 (4)
O27—Mo7—O3—Mo11	101.7 (2)	O69—Mo22—O42—Mo16	1.5 (2)
O27—Mo7—O3—P1	−33.3 (4)	O69—Mo22—O42—Mo17	90.6 (2)
O27—Mo7—O15—Mo3	31.2 (7)	O69—Mo22—O42—P2	−133.3 (4)
O27—Mo7—O26—Mo6	30.7 (8)	O69—Mo22—O70—Mo17	−69.1 (4)
O27—Mo7—O32—Mo11	−86.9 (4)	O69—Mo22—O75—Mo24	126.2 (8)
O27—Mo8—O4—Mo9	−173.8 (2)	O69—Mo22—O76—Mo23	−24.1 (12)
O27—Mo8—O4—Mo12	−84.5 (2)	O70—Mo17—O42—Mo16	90.5 (2)
O27—Mo8—O4—P1	50.8 (4)	O70—Mo17—O42—Mo22	1.44 (19)
O27—Mo8—O16—Mo3	−28.3 (7)	O70—Mo17—O42—P2	−134.7 (4)
O27—Mo8—O28—Mo9	4.5 (8)	O70—Mo17—O53—Mo14	−26.3 (11)
O27—Mo8—O33—Mo12	83.9 (4)	O70—Mo17—O64—Mo16	−68.7 (4)
O28—Mo8—O4—Mo9	2.6 (2)	O70—Mo17—O65—Mo18	126.7 (7)
O28—Mo8—O4—Mo12	91.8 (2)	O70—Mo22—O42—Mo16	−90.7 (2)
O28—Mo8—O4—P1	−132.9 (4)	O70—Mo22—O42—Mo17	−1.5 (2)
O28—Mo8—O16—Mo3	126.0 (7)	O70—Mo22—O42—P2	134.5 (4)
O28—Mo8—O27—Mo7	−63.3 (10)	O70—Mo22—O69—Mo16	70.7 (4)
O28—Mo8—O33—Mo12	−73.2 (4)	O70—Mo22—O75—Mo24	64.2 (10)
O28—Mo9—O4—Mo8	−2.43 (19)	O70—Mo22—O76—Mo23	−122.3 (7)
O28—Mo9—O4—Mo12	−91.8 (2)	O71—Mo18—O43—Mo19	−90.8 (2)
O28—Mo9—O4—P1	133.2 (4)	O71—Mo18—O43—Mo23	−1.0 (2)
O28—Mo9—O11—Mo1	−130.7 (7)	O71—Mo18—O43—P2	133.6 (4)
O28—Mo9—O23—Mo4	22.8 (10)	O71—Mo18—O54—Mo14	65.9 (11)
O28—Mo9—O34—Mo12	69.2 (4)	O71—Mo18—O65—Mo17	−123.5 (7)
O29—Mo4—O2—Mo5	−90.7 (2)	O71—Mo18—O66—Mo19	71.8 (4)
O29—Mo4—O2—Mo10	−1.49 (19)	O71—Mo23—O43—Mo18	0.9 (2)
O29—Mo4—O2—P1	133.5 (4)	O71—Mo23—O43—Mo19	90.5 (2)
O29—Mo4—O12—Mo1	27.3 (10)	O71—Mo23—O43—P2	−133.8 (4)
O29—Mo4—O23—Mo9	−128.6 (7)	O71—Mo23—O72—Mo19	−69.3 (4)
O29—Mo4—O24—Mo5	68.9 (3)	O71—Mo23—O76—Mo22	127.4 (8)
O29—Mo10—O2—Mo4	1.6 (2)	O71—Mo23—O77—Mo24	−24.8 (12)
O29—Mo10—O2—Mo5	90.6 (2)	O72—Mo19—O43—Mo18	91.0 (2)
O29—Mo10—O2—P1	−132.4 (4)	O72—Mo19—O43—Mo23	1.40 (19)
O29—Mo10—O30—Mo5	−71.9 (4)	O72—Mo19—O43—P2	−133.8 (4)
O29—Mo10—O35—Mo12	125.2 (6)	O72—Mo19—O55—Mo15	−21.7 (11)
O29—Mo10—O36—Mo11	−66.9 (9)	O72—Mo19—O66—Mo18	−69.4 (4)
O30—Mo5—O2—Mo4	90.6 (2)	O72—Mo19—O67—Mo20	127.3 (8)
O30—Mo5—O2—Mo10	1.8 (2)	O72—Mo23—O43—Mo18	−91.1 (2)
O30—Mo5—O2—P1	−135.0 (4)	O72—Mo23—O43—Mo19	−1.5 (2)
O30—Mo5—O13—Mo2	−70.4 (9)	O72—Mo23—O43—P2	134.2 (4)
O30—Mo5—O24—Mo4	−72.4 (4)	O72—Mo23—O71—Mo18	72.0 (4)
O30—Mo5—O25—Mo6	124.2 (7)	O72—Mo23—O76—Mo22	65.5 (10)
O30—Mo10—O2—Mo4	−90.7 (2)	O72—Mo23—O77—Mo24	−123.3 (7)
O30—Mo10—O2—Mo5	−1.63 (19)	O73—Mo20—O44—Mo21	−90.8 (2)
O30—Mo10—O2—P1	135.4 (4)	O73—Mo20—O44—Mo24	−1.5 (2)
O30—Mo10—O29—Mo4	69.1 (4)	O73—Mo20—O44—P2	133.7 (5)
O30—Mo10—O35—Mo12	26.1 (10)	O73—Mo20—O56—Mo15	65.5 (10)
O30—Mo10—O36—Mo11	−129.3 (7)	O73—Mo20—O67—Mo19	−122.0 (7)
O31—Mo6—O3—Mo7	−90.6 (2)	O73—Mo20—O68—Mo21	70.2 (4)
O31—Mo6—O3—Mo11	−1.7 (2)	O73—Mo24—O44—Mo20	1.4 (2)
O31—Mo6—O3—P1	133.4 (4)	O73—Mo24—O44—Mo21	91.4 (2)
O31—Mo6—O14—Mo2	26.8 (11)	O73—Mo24—O44—P2	−134.8 (4)
O31—Mo6—O25—Mo5	−127.5 (7)	O73—Mo24—O74—Mo21	−68.6 (4)
O31—Mo6—O26—Mo7	69.6 (4)	O73—Mo24—O75—Mo22	−23.0 (11)
O31—Mo11—O3—Mo6	1.9 (2)	O73—Mo24—O77—Mo23	126.8 (8)
O31—Mo11—O3—Mo7	91.4 (2)	O74—Mo21—O44—Mo20	90.6 (2)
O31—Mo11—O3—P1	−133.5 (4)	O74—Mo21—O44—Mo24	0.9 (2)
O31—Mo11—O32—Mo7	−70.9 (4)	O74—Mo21—O44—P2	−132.8 (4)
O31—Mo11—O36—Mo10	124.4 (7)	O74—Mo21—O51—Mo13	−23.0 (12)
O31—Mo11—O37—Mo12	−65.7 (10)	O74—Mo21—O63—Mo16	127.0 (8)
O32—Mo7—O3—Mo6	90.8 (2)	O74—Mo21—O68—Mo20	−68.2 (4)
O32—Mo7—O3—Mo11	1.9 (2)	O74—Mo24—O44—Mo20	−91.0 (2)
O32—Mo7—O3—P1	−133.1 (4)	O74—Mo24—O44—Mo21	−1.0 (2)
O32—Mo7—O15—Mo3	−68.6 (10)	O74—Mo24—O44—P2	132.8 (4)
O32—Mo7—O26—Mo6	−72.9 (4)	O74—Mo24—O73—Mo20	71.4 (4)
O32—Mo7—O27—Mo8	123.7 (7)	O74—Mo24—O75—Mo22	−122.1 (7)
O32—Mo11—O3—Mo6	−91.3 (2)	O74—Mo24—O77—Mo23	66.0 (10)
O32—Mo11—O3—Mo7	−1.8 (2)	O75—Mo22—O42—Mo16	84.6 (2)
O32—Mo11—O3—P1	133.3 (4)	O75—Mo22—O42—Mo17	173.8 (2)
O32—Mo11—O31—Mo6	68.4 (4)	O75—Mo22—O42—P2	−50.1 (4)
O32—Mo11—O36—Mo10	22.4 (11)	O75—Mo22—O69—Mo16	−86.6 (4)
O32—Mo11—O37—Mo12	−128.8 (7)	O75—Mo22—O70—Mo17	−8.2 (8)
O33—Mo8—O4—Mo9	−89.9 (2)	O75—Mo22—O76—Mo23	31.7 (7)
O33—Mo8—O4—Mo12	−0.62 (19)	O75—Mo24—O44—Mo20	169.7 (2)
O33—Mo8—O4—P1	134.7 (4)	O75—Mo24—O44—Mo21	−100.3 (2)
O33—Mo8—O16—Mo3	26.6 (11)	O75—Mo24—O44—P2	33.5 (4)
O33—Mo8—O27—Mo7	−127.8 (7)	O75—Mo24—O73—Mo20	−30.3 (8)
O33—Mo8—O28—Mo9	67.8 (4)	O75—Mo24—O74—Mo21	86.1 (4)
O33—Mo12—O4—Mo8	0.7 (2)	O75—Mo24—O77—Mo23	−32.2 (7)
O33—Mo12—O4—Mo9	89.7 (2)	O76—Mo22—O42—Mo16	170.1 (2)
O33—Mo12—O4—P1	−135.1 (4)	O76—Mo22—O42—Mo17	−100.7 (2)
O33—Mo12—O34—Mo9	−72.1 (4)	O76—Mo22—O42—P2	35.3 (4)
O33—Mo12—O35—Mo10	−71.2 (9)	O76—Mo22—O69—Mo16	−30.0 (8)
O33—Mo12—O37—Mo11	124.2 (7)	O76—Mo22—O70—Mo17	85.9 (4)
O34—Mo9—O4—Mo8	90.8 (2)	O76—Mo22—O75—Mo24	−32.9 (7)
O34—Mo9—O4—Mo12	1.4 (2)	O76—Mo23—O43—Mo18	84.3 (2)
O34—Mo9—O4—P1	−133.5 (4)	O76—Mo23—O43—Mo19	174.0 (2)
O34—Mo9—O11—Mo1	−67.3 (9)	O76—Mo23—O43—P2	−50.3 (4)
O34—Mo9—O23—Mo4	124.5 (6)	O76—Mo23—O71—Mo18	−85.8 (4)
O34—Mo9—O28—Mo8	−70.2 (4)	O76—Mo23—O72—Mo19	−8.4 (8)
O34—Mo12—O4—Mo8	−90.3 (2)	O76—Mo23—O77—Mo24	31.4 (7)
O34—Mo12—O4—Mo9	−1.29 (19)	O77—Mo23—O43—Mo18	170.1 (2)
O34—Mo12—O4—P1	133.9 (4)	O77—Mo23—O43—Mo19	−100.2 (2)
O34—Mo12—O33—Mo8	70.9 (4)	O77—Mo23—O43—P2	35.5 (4)
O34—Mo12—O35—Mo10	−129.6 (6)	O77—Mo23—O71—Mo18	−28.8 (8)
O34—Mo12—O37—Mo11	25.0 (11)	O77—Mo23—O72—Mo19	87.0 (4)
O35—Mo10—O2—Mo4	100.2 (2)	O77—Mo23—O76—Mo22	−32.6 (8)
O35—Mo10—O2—Mo5	−170.8 (2)	O77—Mo24—O44—Mo20	84.3 (2)
O35—Mo10—O2—P1	−33.8 (4)	O77—Mo24—O44—Mo21	174.3 (2)
O35—Mo10—O29—Mo4	−86.6 (4)	O77—Mo24—O44—P2	−51.9 (4)
O35—Mo10—O30—Mo5	29.4 (8)	O77—Mo24—O73—Mo20	−86.3 (4)
O35—Mo10—O36—Mo11	30.4 (7)	O77—Mo24—O74—Mo21	−9.1 (9)
O35—Mo12—O4—Mo8	−173.1 (2)	O77—Mo24—O75—Mo22	32.2 (7)
O35—Mo12—O4—Mo9	−84.1 (2)	O78—Mo22—O42—Mo16	−22.0 (14)
O35—Mo12—O4—P1	51.1 (4)	O78—Mo22—O42—Mo17	67.2 (14)
O35—Mo12—O33—Mo8	13.4 (8)	O78—Mo22—O42—P2	−156.7 (12)
O35—Mo12—O34—Mo9	86.3 (4)	O78—Mo22—O69—Mo16	173.2 (4)
O35—Mo12—O37—Mo11	−30.5 (7)	O78—Mo22—O70—Mo17	−167.1 (4)
O36—Mo10—O2—Mo4	−173.8 (2)	O78—Mo22—O75—Mo24	−136.8 (7)
O36—Mo10—O2—Mo5	−84.7 (2)	O78—Mo22—O76—Mo23	132.2 (7)
O36—Mo10—O2—P1	52.3 (4)	O79—Mo23—O43—Mo18	−16.7 (13)
O36—Mo10—O29—Mo4	8.1 (8)	O79—Mo23—O43—Mo19	73.0 (13)
O36—Mo10—O30—Mo5	85.4 (4)	O79—Mo23—O43—P2	−151.3 (11)
O36—Mo10—O35—Mo12	−29.0 (6)	O79—Mo23—O71—Mo18	175.2 (4)
O36—Mo11—O3—Mo6	101.5 (2)	O79—Mo23—O72—Mo19	−166.8 (4)
O36—Mo11—O3—Mo7	−169.0 (2)	O79—Mo23—O76—Mo22	−135.9 (8)
O36—Mo11—O3—P1	−33.9 (4)	O79—Mo23—O77—Mo24	130.7 (7)
O36—Mo11—O31—Mo6	−86.4 (4)	O80—Mo24—O44—Mo20	−19.2 (13)
O36—Mo11—O32—Mo7	33.3 (8)	O80—Mo24—O44—Mo21	70.8 (12)
O36—Mo11—O37—Mo12	30.8 (7)	O80—Mo24—O44—P2	−155.4 (11)
O37—Mo11—O3—Mo6	−173.8 (2)	O80—Mo24—O73—Mo20	173.5 (4)
O37—Mo11—O3—Mo7	−84.3 (2)	O80—Mo24—O74—Mo21	−166.9 (4)
O37—Mo11—O3—P1	50.8 (4)	O80—Mo24—O75—Mo22	132.7 (7)
O37—Mo11—O31—Mo6	7.0 (8)	O80—Mo24—O77—Mo23	−136.1 (7)
O37—Mo11—O32—Mo7	86.2 (4)	O1S—C11—C12—C13	75.0 (13)
O37—Mo11—O36—Mo10	−29.7 (7)	O8S—C81—C82—C83	54 (2)
O37—Mo12—O4—Mo8	100.7 (2)	N1—C11—C12—C13	−99.8 (11)
O37—Mo12—O4—Mo9	−170.3 (2)	N8—C81—C82—C83	−122.6 (15)
O37—Mo12—O4—P1	−35.1 (4)	C14—N1—C11—O1S	−0.1 (15)
O37—Mo12—O33—Mo8	−85.2 (4)	C14—N1—C11—C12	174.9 (9)
O37—Mo12—O34—Mo9	29.9 (8)	C15—N1—C11—O1S	−178.3 (9)
O37—Mo12—O35—Mo10	30.3 (6)	C15—N1—C11—C12	−3.3 (16)
O38—Mo10—O29—Mo4	167.7 (4)	C84—N8—C81—O8S	2.7 (16)
O38—Mo10—O30—Mo5	−175.1 (4)	C84—N8—C81—C82	179.7 (10)
O38—Mo10—O35—Mo12	−129.0 (6)	C85—N8—C81—O8S	177.6 (10)
O38—Mo10—O36—Mo11	133.3 (7)	C85—N8—C81—C82	−5.5 (17)
O39—Mo11—O31—Mo6	167.5 (4)	O2S—C21—C22—C23	85.5 (19)
O39—Mo11—O32—Mo7	−174.7 (4)	O11S—C111—C112—C113	−3.2 (15)
O39—Mo11—O36—Mo10	−129.1 (7)	N2—C21—C22—C23	−91.5 (18)
O39—Mo11—O37—Mo12	133.5 (7)	N11—C111—C112—C113	−177.7 (9)
O40—Mo12—O4—Mo8	−71.4 (12)	C115—N11—C111—O11S	177.6 (10)
O40—Mo12—O4—Mo9	17.6 (12)	C115—N11—C111—C112	−7.2 (15)
O40—Mo12—O4—P1	152.8 (11)	C24—N2—C21—O2S	2.7 (18)
O40—Mo12—O33—Mo8	167.7 (3)	C24—N2—C21—C22	179.8 (10)
O40—Mo12—O34—Mo9	−174.1 (4)	C25—N2—C21—O2S	−178.5 (12)
O40—Mo12—O35—Mo10	134.4 (6)	C25—N2—C21—C22	−1.4 (17)
O40—Mo12—O37—Mo11	−130.4 (7)	C114—N11—C111—O11S	2.1 (14)
O41—Mo13—O48—Mo15	−2.0 (3)	C114—N11—C111—C112	177.4 (9)
O41—Mo13—O49—Mo14	2.5 (3)	O3S—C31—C32—C33	62.5 (11)
O41—Mo13—O51—Mo21	54.9 (7)	O7S—C71—C72—C73	83.7 (11)
O41—Mo13—O52—Mo16	−53.4 (7)	N3—C31—C32—C33	−118.9 (10)
O41—Mo14—O49—Mo13	−2.5 (3)	N7—C71—C72—C73	−91.6 (11)
O41—Mo14—O50—Mo15	1.8 (3)	C34—N3—C31—O3S	1.4 (13)
O41—Mo14—O53—Mo17	55.9 (7)	C34—N3—C31—C32	−177.2 (8)
O41—Mo14—O54—Mo18	−50.2 (8)	C35—N3—C31—O3S	178.7 (8)
O41—Mo15—O48—Mo13	2.0 (3)	C35—N3—C31—C32	0.1 (14)
O41—Mo15—O50—Mo14	−1.9 (3)	C74—N7—C71—O7S	2.3 (14)
O41—Mo15—O55—Mo19	54.0 (6)	C74—N7—C71—C72	177.6 (8)
O41—Mo15—O56—Mo20	−49.4 (7)	C75—N7—C71—O7S	−177.2 (9)
O41—P2—O42—Mo16	66.1 (4)	C75—N7—C71—C72	−1.9 (14)
O41—P2—O42—Mo17	−53.6 (5)	O4S—C41—C42—C43	1.1 (14)
O41—P2—O42—Mo22	−174.4 (3)	O5S—C51—C52—C53	90.0 (12)
O41—P2—O43—Mo18	67.3 (4)	N4—C41—C42—C43	178.4 (9)
O41—P2—O43—Mo19	−53.0 (5)	N5—C51—C52—C53	−96.7 (12)
O41—P2—O43—Mo23	−173.5 (3)	C44—N4—C41—O4S	0.8 (15)
O41—P2—O44—Mo20	65.2 (5)	C44—N4—C41—C42	−176.6 (9)
O41—P2—O44—Mo21	−56.1 (5)	C45—N4—C41—O4S	−177.9 (10)
O41—P2—O44—Mo24	−174.2 (3)	C45—N4—C41—C42	4.6 (15)
O42—Mo16—O52—Mo13	58.6 (7)	C54—N5—C51—O5S	175.5 (9)
O42—Mo16—O63—Mo21	−50.0 (7)	C54—N5—C51—C52	2.4 (16)
O42—Mo16—O64—Mo17	−2.1 (3)	C55—N5—C51—O5S	5.2 (15)
O42—Mo16—O69—Mo22	2.4 (3)	C55—N5—C51—C52	−167.9 (9)
O42—Mo17—O53—Mo14	−50.8 (7)	O6S—C61—C62—C63	−94.5 (10)
O42—Mo17—O64—Mo16	2.1 (3)	N6—C61—C62—C63	83.8 (11)
O42—Mo17—O65—Mo18	55.3 (7)	C64—N6—C61—O6S	178.9 (8)
O42—Mo17—O70—Mo22	−2.4 (3)	C64—N6—C61—C62	0.6 (14)
O42—Mo22—O69—Mo16	−2.4 (3)	C65—N6—C61—O6S	1.2 (13)
O42—Mo22—O70—Mo17	2.4 (3)	C65—N6—C61—C62	−177.2 (8)
O42—Mo22—O75—Mo24	53.9 (7)	O9S—C91—C92—C93	75.1 (14)
O42—Mo22—O76—Mo23	−50.2 (7)	N9—C91—C92—C93	−104.8 (13)
O42—P2—O41—Mo13	−52.9 (5)	C94—N9—C91—O9S	1.1 (16)
O42—P2—O41—Mo14	64.4 (5)	C94—N9—C91—C92	−179.0 (10)
O42—P2—O41—Mo15	−173.9 (3)	C95—N9—C91—O9S	−176.3 (9)
O42—P2—O43—Mo18	−53.5 (5)	C95—N9—C91—C92	3.6 (17)
O42—P2—O43—Mo19	−173.8 (3)	O10S—C101—C102—C103	−94.0 (12)
O42—P2—O43—Mo23	65.7 (5)	N10—C101—C102—C103	84.3 (12)
O42—P2—O44—Mo20	−173.4 (3)	C104—N10—C101—O10S	3.4 (13)
O42—P2—O44—Mo21	65.3 (5)	C104—N10—C101—C102	−174.9 (9)
O42—P2—O44—Mo24	−52.8 (5)	C105—N10—C101—O10S	176.2 (8)
O43—Mo18—O54—Mo14	54.9 (7)	C105—N10—C101—C102	−2.2 (14)
O43—Mo18—O65—Mo17	−50.6 (7)		

### Hydrogen-bond geometry (Å, º)

**Table:**

*D*—H···*A*	*D*—H	H···*A*	*D*···*A*	*D*—H···*A*
O1*S*—H1*S*···O8*S*	1.22	1.22	2.440 (10)	179
O11*S*—H2*S*···O2*S*	1.21	1.22	2.426 (11)	179
O3*S*—H3*S*···O7*S*	1.21	1.22	2.429 (9)	180
O4*S*—H4*S*···O5*S*	1.22	1.23	2.450 (10)	180
O6*S*—H6*S*···O8^i^	0.84	1.78	2.606 (8)	170
O9*S*—H9*S*···O9*S*^ii^	1.22	1.22	2.447 (10)	180
O10*S*—H10*S*···O10*S*^iii^	1.22	1.22	2.430 (13)	180

Symmetry codes: (i) *x*, *y*, *z*+1; (ii) −*x*+1, −*y*+1, −*z*; (iii) −*x*, −*y*+1, −*z*+2.
